# *In vivo* labelling resolves distinct temporal, spatial, and functional properties of tumour macrophages, and identifies subset-specific effects of PD-L1 blockade

**DOI:** 10.1158/2326-6066.CIR-24-1233

**Published:** 2025-09-02

**Authors:** Colin Y.C. Lee, Isaac Dean, Nathan Richoz, Zhi Li, Bethany C. Kennedy, Lisa A. Vettore, Youhani Samarakoon, Kathryn L. Gilroy, Tetsuo Hasegawa, Gianluca Carlesso, Scott A. Hammond, Simon J. Dovedi, Owen J. Sansom, Zewen K. Tuong, Timotheus Y.F. Halim, David R. Withers, Menna R. Clatworthy

**Affiliations:** 1 Molecular Immunity Unit, Department of Medicine, Medical Research Council Laboratory of Molecular Biology, University of Cambridge, Cambridge, UK; 2 Cellular Genetics, Wellcome Sanger Institute, Wellcome Genome Campus, Hinxton, Cambridge, UK; 3 Institute of Immunology and Immunotherapy, College of Medical and Dental Sciences, University of Birmingham, Birmingham, UK; 4 NDM Centre for Immuno-Oncology, University of Oxford, Oxford, UK; 5 Cancer Research UK Cambridge Institute, Li Ka Shing Centre, University of Cambridge, Cambridge, UK; 6 Cancer Research UK Scotland Institute, Glasgow, UK; 7 Early Oncology R&D, AstraZeneca, Gaithersburg, USA; 8 Early Oncology R&D, AstraZeneca, Cambridge, UK

## Abstract

Tumour-associated macrophages (TAMs) are a universal feature of cancers but variably influence outcome and treatment responses. Here, we used a photoconvertible mouse to distinguish newly entering, monocyte-derived (md)TAMs that were enriched at the tumour core, from resident-like (r)TAMs that localised with fibroblasts at the tumour–normal interface. The mdTAM pool was highly dynamic and continually replenished by circulating monocytes. Upon tumour entry, these monocytes differentiated down two divergent fate trajectories distinguished by the expression of MHC class II. MHC-II^+^ mdTAMs were functionally distinct from MHC-II^–^ mdTAMs, demonstrating increased capacity for endocytosis and FcγR-mediated phagocytosis, as well as pro-inflammatory cytokine production. Both mdTAM subsets showed reduced expression of inflammatory transcripts and increased expression of PD-L1 with increasing tumour dwell-time. Treatment with anti-PD-L1 skewed mdTAM differentiation towards the MHC-II^+^ fate and attenuated the anti-inflammatory effects of the tumour environment. Anti-PD-L1 enhanced mdTAM–CD4^+^ T-cell interactions, establishing an IFNγ-CXCL9/10-dependent positive feedback loop. Altogether, these data resolve distinct temporal, spatial and functional properties of TAMs, and provide evidence of subset-specific effects of PD-L1 blockade.

## Introduction

Macrophages are abundant in solid tumours through all stages of tumourigenesis, regardless of whether the tumour is ‘hot’ (lymphocyte-rich) or ‘cold’ (lymphocyte-sparse). Their universal presence in tumours represents an attractive target for anticancer therapies, but tumour-associated macrophages (TAMs) are variably associated with prognosis and therapeutic response (1–3). Indeed, the tumour environment corrupts the molecular profile of TAMs to reduce antitumour functionality (4). Conventionally, a binary model of pro-inflammatory antitumourigenic “M1” or anti-inflammatory pro-tumourigenic “M2” macrophages has been described (5), the latter purportedly dominating in most tumours (4,6). However, “M1” and “M2” programmes simultaneously exist in multiple TAM subsets across tumour types (7), and are too simplistic to accurately define their complex spectrum of transcriptional states (8,9). This transcriptional heterogeneity is associated with variable function. Antitumourigenic TAMs may recruit and activate lymphocytes, promote their expansion, or facilitate antibody-dependent responses via Fc-gamma receptors (FcγRs) (10,11). Conversely, TAMs may promote tumourigenesis by supporting tumour invasion, angiogenesis, or the expression of immunosuppressive molecules (3,4,11), including IL-10, TGFβ, and immune checkpoint ligands such as PD-L1, which inhibit cytotoxic T-cell immunosurveillance (12). Tumour cells also express PD-L1, but notably, PD-L1 expression by TAMs specifically correlates with efficacy of immune checkpoint blockade (ICB) therapies (13–15) and is required for responses to anti-PD-L1 in murine cancer models (16).

In homeostasis, macrophages originate from early haematopoietic precursors in the embryonic yolk sac or foetal liver (17). These prenatally-seeded macrophages are maintained by proliferation *in situ* and are variably replaced post-natally by haematopoietic stem cell (HSC)-derived macrophages that differentiate from circulating blood monocytes in an organ-specific manner (18). During inflammation, monocyte recruitment is increased, boosting the local macrophage pool (19). In murine models of breast, lung and colorectal carcinomas, TAMs are thought to arise from circulating monocytes (20–22), and may proliferate following tumour entry (21). However, other studies have demonstrated that embryonic-derived tissue-resident macrophages also sustain the TAM pool (23,24). How ontogeny relates to TAM phenotype and function remains incompletely understood.

To harness TAMs to enhance antitumour responses, a better understanding of TAM heterogeneity and how different TAM subsets vary in terms of function, susceptibility to the inhibitory tumour environment, and responses to ICB is required. Here, we performed spatio-dynamic photo-labelling of tumour immune cells in Kaede-transgenic mice (25), combined with single-cell RNA sequencing (scRNA-seq), to directly assess temporal changes to the TAM compartment in real-time. We found two major populations: resident-like (r)TAMs that interact with fibroblasts at the tumour–normal interface and monocyte-derived (md)TAMs that are continuously and rapidly replenished by circulating precursors. mdTAMs progressed along two fate trajectories within tumours, with distinct phenotypes and functions. Newly-infiltrating TAMs were most affected by PD-L1 blockade, which attenuated the rapid loss of inflammatory gene expression upon monocyte entry into the tumour and established self-potentiating IFNγ-driven responses between TAMs and CD4^+^ T cells. Altogether, our data provide critical insights into TAM heterogeneity in space and time, and delineate subtype-specific responses to ICB therapy.

## Materials and Methods

### Mice

C57BL/6, BALB/c, transgenic C57BL/6 Kaede and transgenic BALB/c Kaede mice were maintained and bred at the University of Birmingham Biomedical Services Unit. Mice were sacrificed between the ages of 8 and 14 weeks. All animal experiments were conducted in accordance with Home Office guidelines and were approved by the University of Birmingham Animal Welfare and Ethical Review Body. Mice were housed at 21°C, 55% humidity, with 12 h light-dark cycles in individually ventilated caging with environmental enrichment of plastic houses plus paper bedding.

### Mouse subcutaneous tumour model

MC38 (obtained from Kerafast, catalogue no. ENH204-FP), CT26 (kindly provided by Professor Tim Elliot, University of Oxford, Oxford, UK), and MC38-Ova (provided by AstraZeneca) murine colon adenocarcinoma cells were cultured in DMEM (MC38; Gibco) or RPMI (CT26; Gibco), supplemented with 2mM L-glutamine (Thermo Fisher Scientific), 10% FBS (F9665; Sigma-Aldrich), and 1% penicillin-streptomycin (Sigma-Aldrich), hereafter referred to as complete DMEM or complete RPMI (cRPMI), at 37°C with 5% CO_2_. Cells grown in the log-phase were harvested and resuspended to 2.5 × 10^6^ cells/ml in Dulbecco’s PBS (Sigma-Aldrich) for tumour injection. Mycoplasma testing was performed every 3 to 6 months using the PlasmoTest™ (Invitrogen) or MycoStrip™ (Invitrogen) kits following the manufacturer’s instructions. Cells were passaged at least twice per week; all cells used underwent less than 15 passages. Cell lines were not re-authenticated.

2.5 × 10^5^ tumour cells in 100 μl were subcutaneously injected into mice in the pre-shaved left flank area under anaesthesia via 2% gaseous isoflurane. Tumour size was measured periodically with a digital Vernier calliper, and the volume was calculated using the formula V = 0.5 × a × b^2^ in cubic millimetres, where a and b are the long and short diameters of the tumour. Mice were sacrificed on day 13, 14, 15, or 16 (5h, 24h, 48h, 72h post-photoconversion respectively, where performed) and tumours were obtained for analysis.

### Injection of anti-PD-L1

Anti-PD-L1 mouse IgG1 (Clone 80, SP16-260; AstraZeneca) or NIP228 isotype control mouse IgG1 (SP16-017; Astra Zeneca) were administered on day 7, 10, and 13 after tumour inoculation by intraperitoneal injection. Each dose consisted of 200 μg antibodies diluted in 200 μl PBS (10 mg/kg body weight). Tumour volume was measured on day 7, 10, 13 and the experiment endpoint.

### Labelling of tumour compartment by photoconversion

Photoconversion was performed as previously described (25). Briefly, on day 13 following tumour injection, the subcutaneous tumour was exposed to a 405-nm wavelength focussed LED light (Dymax BlueWave QX4 outfitted with 8mm focussing length, DYM41572; Intertronics) for 180 seconds, with a 5-second break every 20 seconds, at a fixed distance of 1 cm. Black cardboard was used to shield the remainder of the mouse. This effectively resulted in complete conversion (mean 98%, minimum 95%) of host cells within the tumour from the default green fluorescence of the Kaede protein (Kaede-green) to the altered red fluorescence (Kaede-red), and cells in the draining lymph node (dLN) were fully protected from tumour photoconversion (25). Converted cells expressed Kaede-red fluorescence, but they also retained Kaede-green signal. Overall, this enabled the discrimination of newly infiltrating (Kaede-green) and resident (Kaede-red) cell populations in the tumour by their fluorescence profile. Moreover, using intravenous administration of anti-CD45 prior to culling, we previously showed that majority of cells (>95%) were in tumour tissue and were not intravascular contaminants (25).

### Immunofluorescence microscopy

Tumours were dissected and fixed in 1% paraformaldehyde (Electron Microscopy Services) for 24h at 4°C followed by 12h in 30% sucrose in PBS. 20μm sections were permeabilized and blocked in 0.1M TRIS, containing 0.1% Triton (Sigma), 1% normal mouse serum (Thermo Fisher Scientific), 1% normal rat serum (Thermo Fisher Scientific) and 1% BSA (R&D Systems). Samples were stained for 2h at room temperature (RT) in a humid chamber with the appropriate antibodies, listed in Supplementary table S1, washed 3 times in PBS and mounted in Fluoromount-G® (Southern Biotech). Images were acquired using a TCS SP8 (Leica microsystems, Milton Keynes, UK) confocal microscope. Raw imaging data were processed using Imaris v9.7.2 (Bitplane).

Iterative staining of sections was performed as previously described (26). Samples were prepared and stained as detailed above. Following acquisition, the coverslips were removed, and slides were washed 3 times in PBS to remove residual mounting medium. Bleaching of the fluorochromes was achieved by submerging the slide in a 1mg/mL solution of lithium borohydride in water (Acros Organics) for 15 minutes at room temperature. The slides were then washed 3 times in PBS prior to staining with a different set of antibodies. The process was repeated twice. Raw imaging data were processed using Imaris using CD31 as fiducial for the alignment of acquired images.

For quantification of fluorescence microscopy, imaging data from Imaris were further analysed using QuPath (27). Hoechst nuclear staining was first used to perform automated cell detection with the nucleus diameter setting at 3-10 μm. Thereafter, detections were manually annotated using antibody staining of cellular markers and morphology to identify 100 cells each for cell types of interest. The manual annotations were used to train a semi-automated random trees object classifier using nuclear and cellular morphology, and available fluorescence staining intensities. The classification output and centroid positions for all cell detections were exported for further analysis in R (v4.1.2). For heatmaps of F4/80 and CD11b fluorescence, tumour images were divided into 10-pixel grids (∼5μm) and pixel intensity was averaged across each grid, before gaussian kernel density estimation was applied to analyse spatial distribution of staining. The tumour core was defined as >100 μm from the tumour edges. For CD4^+^ T-cell quantification, 500μm x 500μm grids were randomly selected without overlap from the tumour core, 10 grids from isotype control-treated tumours and 5 from anti-PD-L1-treated tumours which were smaller, and cells were counted per grid.

### Tissue dissociation

Tumours were cut into small pieces using surgical scissors, and incubated with 1 mg/mL collagenase D (Roche) and 0.1 mg/mL DNase I (Roche) in a volume of 1.2 ml RPMI media at 37°C on a thermomixer (Eppendorf) for 20 min. Subsequently, the sample was filtered through a 70 μm strainer to remove undigested tissue debris. Next, dead cells were removed using Dead Cell Removal Kit and LS Columns (Miltenyi Biotec) or EasySep Dead Cell Removal kit (Stemcell technologies), according to the manufacturers’ instructions. Thereafter, cells were centrifuged at 400 *g* at 4°C for 5 min and resuspended in FACS staining buffer (2% FBS; 2mM EDTA in PBS) for flow cytometry.

### *Ex vivo* cultures

Tumours were digested as described above. Samples were centrifuged in a 30% Percoll (Sigma-Aldrich) gradient for 20 minutes to remove tissue debris, prior to dead cell removal using the EasySep kit (Stemcell technologies). Individual tumours were resuspended in pre-warmed cRPMI and split equally into multiple wells for paired stimulation *ex vivo*, to enable intra-tumour comparisons. *Ex vivo* cultures were performed at 37°C in 48-well plates with 300 μL per well containing 10^7^ cells / mL of tumour cell suspensions. For assessment of antigen presentation, Eα(52–68) peptide at 1 μg/mL concentration (Anaspec) or PBS control were added for 2h before staining with biotinylated YAe antibody (eBioY-Ae, Thermo Fisher Scientific). For ovalbumin (Ova) or Ova-immune complex (Ova-IC) stimulation, AF647-conjugated Ova (Invitrogen) was first mixed with unconjugated Ova (InvivoGen) at a 1:4 ratio to a final concentration of 1 mg/mL. Large ICs were prepared by incubating Ova mixture with rabbit polyclonal anti-Ova antiserum (3.7 mg/mL; C-6534, Sigma-Aldrich) at a 1:5 molar ratio for 45 minutes in a 37°C water bath. ICs were washed twice (centrifuged at 10,000 rpm for 60s, discarding supernatant), prior to use. 0.5 μg of Ova or equivalent IgG-complexed Ova was added per well for 6h. For assessment of cytokine production, isotype control or anti-PD-L1 (25 μg/mL each), or recombinant murine IFNγ (10 ng/mL, Peprotech) was added for 8h. GolgiPlug protein transport inhibitor (BD) was added 4h prior to flow cytometry staining to enhance cytokine detection. Cells were collected, culture plates washed twice with ice-cold PBS supplemented with 2mM EDTA and 10% FBS, and filtered before downstream application.

### Flow cytometry

Cell suspensions were subjected to Fc block with anti-CD16/32 (clone 2.4G2, BioLegend) diluted in FACS staining buffer on ice for 15 min before staining with a Live/Dead stain and surface markers, listed in Supplementary table S1, diluted in FACS staining buffer or Brilliant Stain buffer (BD) on ice for 30 min. Where applicable, streptavidin-fluorophore secondary staining was performed on ice for 20 minutes. Where applicable, cells were fixed with Cytofix/Cytoperm solution (BD) for 20 min and stained for intracellular markers diluted in BD Perm/Wash buffer (BD) at 4°C overnight. 1 × 10^4^ counting beads (Spherotech) were added to stained samples at the final step, to calculate absolute cell numbers. Data were acquired on the LSR Fortessa X-20 (BD) using FACSDiva v8.0.2 software (BD) or CytoFLEX (Beckman Coulter) using CytExpert v2.5 (Beckman Coulter) and analysed with FlowJo v10.8.1 (BD).

### Single-cell isolation

Age-matched female Kaede C57BL/6 mice inoculated with MC38-Ova tumours, treated with anti-PD-L1, and photoconverted on day 13 were used, as described above. Mice with tumours of similar sizes were collected 48h after tumour photoconversion. After tumour digestion, as described above, cell suspensions were stained for CD45 BV786, TER119 PE-Cy7, CD11b BV421, NK1.1 BV650, Live/dead APC-Cy7 on ice for 30 min (Supplementary table S1). Subsequently, cells were centrifuged at 400 *g* at 4°C for 5 min and resuspended in FACS staining buffer for sorting. Tumour-infiltrating myeloid cells (Live CD45^+^TER119^–^Kaede^+^CD11b^+^NK1.1^–^) or tumour-infiltrating lymphocytes (Live CD45^+^TER119^–^Kaede^+^CD11b^–/low^NK1.1^low/hi^) from anti-PD-L1 or isotype control-treated tumours were sorted with a FACS Aria II Cell Sorter (BD) into two groups per cell type, based on the presence or absence of Kaede-red signal. CD45^+^ cells were only sorted to myeloid or tumour-infiltrating lymphocyte (TIL) (CD11b^+^ or CD11b^–/low^) fractions to ensure appropriate representation of various cell types in the scRNA-seq data. All single-cell transcriptomes were combined at the analysis stage, to ensure all CD45^+^ immune cells, regardless of surface CD11b or NK1.1 expression, were represented in the final analysis and cells were annotated only based on their gene expression profiles.

### Bulk RNA-seq library construction and sequencing

MC38-Ova tumours were collected 24h or 72h after photoconversion and processed as described above. Live CD45^+^TER119^–^Kaede^+^CD11b^+^NK1.1^–^ cells were isolated by FACS sorting, and RNA was extracted using a Qiacube with the RNAmini kit (Qiagen). Libraries were prepared using the SMARTer stranded total RNAseq mammalian pico input kit (Takara) according to the manufacturer’s instructions. 5 ng of total RNA was used for production of libraries. Libraries were pooled at an equimolar concentration and sequencing was performed using a Novaseq 6000 (Illumina) on a 2 × 150 bp sequencing run.

### Processing and analysis of bulk RNA-seq

Pooled libraries were demultiplexed using Casava (Illumina). Fastq files from sequencing libraries were trimmed of the first 3 nucleotides of the R1 strand. Contaminating adaptor sequences and poor-quality bases were removed using Trim Galore (Babraham bioinformatics). Libraries were only trimmed for quality. Quality of resulting files were assessed by FastQC and aligned to the mm10 genome using HISAT2, followed by gene-level quantification using the Featurecount function from the Rsubread package in R. Analysis was performed using the standard DESeq2 (v1.34) workflow and gene set enrichment analysis (GSEA), implemented in fgsea (v1.24), was used for pathway analysis, using the Wald statistic as the pre-rank metric.

### Single-cell library construction and sequencing

Single-cell gene expression libraries from were prepared from FACS-sorted populations of MC38-Ova tumour myeloid cells or lymphocytes, as described above, using the Chromium Controller and Chromium Single Cell 3’ GEM Reagent Kits v3 (10x genomics, Inc.) according to the manufacturer’s protocol. The resulting sequencing libraries comprised of standard Illumina paired-end constructs flanked with P5 and P7 sequences. The 16 bp 10x barcode and 10 bp unique molecular identifier (UMI) were encoded in read 1, while read 2 was used to sequence the cDNA fragment. Sample index sequences were incorporated as the i7 index read. Paired-end sequencing (2 × 150 bp) was performed on a NovaSeq 6000 (Illumina). The resulting.bcl sequence data were processed for QC purposes using bcl2fastq software (v2.20.0.422) and the resulting fastq files were assessed using FastQC (v0.11.3), FastqScreen (v0.9.2) and FastqStrand (v0.0.5) prior to alignment and processing with the CellRanger (v6.1.2) pipeline.

### Processing of scRNA-seq data

Single-cell gene expression data from CellRanger count output (filtered features, barcodes, and matrices) were analysed using the Scanpy (28) (v1.8.2) workflow. Raw count data from the myeloid and TIL sorts were concatenated. Doublet detection was performed using Scrublet (29) (v0.2.1), with cells from iterative sub-clustering flagged with outlier Scrublet scores labelled as potential doublets. Cells with counts mapped to >6000 or <1000 genes were filtered. The percentage mitochondrial content cut-off was set at <7.5%. Genes detected in fewer than 3 cells were filtered. Total gene counts for each cell were normalised to a target sum of 10^4^ and log1p transformed. Next, highly variable features were selected based on a minimum and maximum mean expression of ≥0.0125 and ≤3 respectively, with a minimum dispersion of 0.5. Total feature counts, mitochondrial percentage, and cell cycle scores, where indicated, were regressed. The number of principal components used for neighbourhood graph construction was set to 30 initially, and subsequently 30 for macrophage and CD4^+^ T-cell subgroup processing. Clustering was performed using the Leiden algorithm with resolution set at 1.5 for initial annotations, but subsequently sub-clustering was performed at lower resolutions (0.7-1.0) for analysis of macrophage and CD4 T cell subsets. Uniform manifold approximation and projection (UMAP, v0.5.1) was used for dimensional reduction and visualisation, with a minimum distance of 0.3, and all other parameters according to the default settings in Scanpy.

### Analysis of scRNA-seq data

Macrophages were subset and re-clustered as described above. Resulting clusters were annotated using canonical marker gene expression, published transcriptomic signatures and Kaede profile. Unless otherwise indicated, log-transformed expression values were used for plotting. Gene set scoring was performed using Scanpy’s tl.score_genes tool. Gene sets were obtained from the Molecular Signature Database (MSigDB) inventory, specifically Hallmark, KEGG, or Gene Ontology (GO), using the R package msigdbr (v7.5.1) or published RNAseq data and signatures as included in the *results* text. Differential gene testing between clusters was performed using the Wilcoxon rank sum test implemented in Scanpy’s tl.rank_genes_groups. Analysis of gene regulatory networks and inference of transcription factor regulon activity and specificity scores were performed using pySCENIC (v0.12.0), the python implementation of SCENIC (30). Network inference using GRNBoost2 and cellular enrichment using AUCell was used, according to the developers’ recommendations.

For trajectory inference, diffusion maps were first used to embed the single-cell gene expression profiles (31). Subsequent analysis was performed independently using partition-based graph abstraction (PAGA) (32) and the Palantir algorithm (33). For Palantir pseudo-time analysis, the differentiation trajectory was rooted in the Kaede-green dominant cluster, mdTAM_1/monocytes, which represents the cellular subset most associated with newly-infiltrating cells, and also had the highest expression of monocyte-associated transcripts, including *Ly6c2* and *Sell*. The specific root cell was selected based on the extrema of diffusion components. No end-points were specified, so the number of trajectories and direction of trajectories were computed without prior bias. Single cells were classified into trajectories based on the trajectory probabilities computed in Palantir, using a cut-off of 0.5. Thereafter, differential expression analysis between trajectories was implemented in tradeSeq (34), using the diffEnd test and pattern test functions. For plotting differential expression between trajectories, cells with a probability of >0.7 for each trajectory were obtained and gene expression values were averaged, and significance testing values were obtained from tradeSeq’s diffEnd test. For plotting of genes or genesets over pseudotime, local estimated scatterplot smoothing (loess) regression was used.

For pathway analysis between Kaede-red versus Kaede-green or anti-PD-L1 versus isotype control groups in the scRNA-seq dataset, a pseudo-bulk approach was first applied to single-cell gene expression data (https://github.com/colin-leeyc/CLpseudobulk), to increase robustness for pathway analysis and overcome limitations associated with differential expression testing on single cells. Briefly, filtered, raw count data (prior to normalisation, transformation, or scaling) from each condition were randomly sorted into artificial replicates, with iterative bootstrapping applied to random sampling (*n*>10). Pseudo-bulked count matrices were normalised using the median-of-ratios method, implemented in DESeq2 (v1.34), and differential gene expression testing was performed using the Wald test. Pre-ranked GSEA was implemented in fgsea (v1.24), using the averaged Wald statistic over *n* >10 iterations of random sorting into pseudo-bulked replicates, as the gene rank metric. Leading edge genes were identified for further analysis.

Gaussian kernel density estimation to compute density of cells in the UMAP embedding was performed using Scanpy’s tl.embedding_density. Differential abundance analysis was performed using Milo. Specifically, the *k*-nearest-neighbour (kNN) graph was constructed with a *k* parameter of 30 and initial random sampling rate of 0.1. Cellular neighbourhoods with constituent cells comprising less than 70% of a previously defined cluster were designated as mixed neighbourhoods. Cell–cell communication analysis was performed using CellChat (35), following the developers’ recommendations and default parameters. Output data was obtained and visualised in R.

Whole-tumour scRNA-seq of orthotopically transplanted *Kras*^G12D/+^ *Trp53*^fl/fl^ *Notch1*^Tg^ (KPN) tumour organoids, *Apc*^fl/fl^ *Kras*^G12D/+^ *Trp53*^fl/fl^ *Tgfbr1*^fl/fl^ (AKPT) tumour organoids, and tamoxifen-induced *Villin-cre*^ERT2^ *Apc*^fl/fl^ murine colon tumours (Research Square preprint doi.org/10.21203/rs.3.rs-6812365/v1) were processed as previously described. Monocytes and macrophages were annotated and subset, followed by integration and label transfer with Scanpy’s tl.ingest, using the MC38 scRNA-seq data as the reference. Transcriptional similarity was assessed by Pearson correlation of the PCA representation of TAM clusters in respective scRNA-seq data.

### Spatial transcriptomics

Visium data (2 independent human colorectal tumour sections; slide IDs V52Y10-310-A1 and V10A13-206-C1) was downloaded from 10X Genomics (36) and analysed using the standard Scanpy (v1.9.3) workflow, using the default SpaceRanger outputs. Spots with fewer <500 counts and percentage mitochondrial content >20% were filtered. Genes detected in fewer than 5 spots were filtered. Expression values plotted are log1p-transformed 10^4^-sum-normalised values. Gene set scoring was performed using Scanpy’s tl.score_genes tool. Genes used for cell type scores were as follows: monocyte/macrophage (*CD68, CCR2, ITGAM, CD14, CX3CR1, CD74, CST3, VCAN, S100A8, CD163, CSF1R*), CD4^+^ T cells (*CD3D, CD3E, CD4, TRAC, TRBC2, CD40LG*). For identification of monocyte/macrophage^+^ spots, *CD68* and *CD14* expression was used; for identification of CD4^+^ T cell^+^ spots, *TRAC* and *CD4* expression was used.

### Statistical analysis

Mice were gender-matched (all female). Tumour growth curves for anti-PD-L1 and isotype control treated groups were analysed using two-way ANOVA and Sidak’s multiple comparisons test and are presented as mean ± SEM. Analysis for RNA sequencing is as described above, including statistical frameworks used. Statistical tests were implemented for two principal purposes; to compare expression values (RNA or protein) between samples, or to compare proportions of defined cell subsets. All experiments were performed with biological replicates, and the specific statistical tests applied are indicated in the figure legends. All statistical tests applied were two-tailed. Paired statistical tests were used where different populations from the same animal were compared, or where biological samples were split to compare effect of different stimulations. Corrections for multiple testing were applied when n>2 comparisons were made. All animal experiments were randomised prior to experimental intervention. Statistical significance is denoted as follows: ns, not significant; *P < 0.05; **P < 0.01; ***P < 0.001; ****P < 0.0001. Violin plot horizontal lines represent the median, 25^th^ and 75^th^ percentile, minima and maxima. Statistical analyses were performed in R (v 4.1.2) or GraphPad Prism (v9.5.0).

## Results

### *In vivo* labelling reveals a rapidly changing myeloid cell landscape

**Figure 1:**
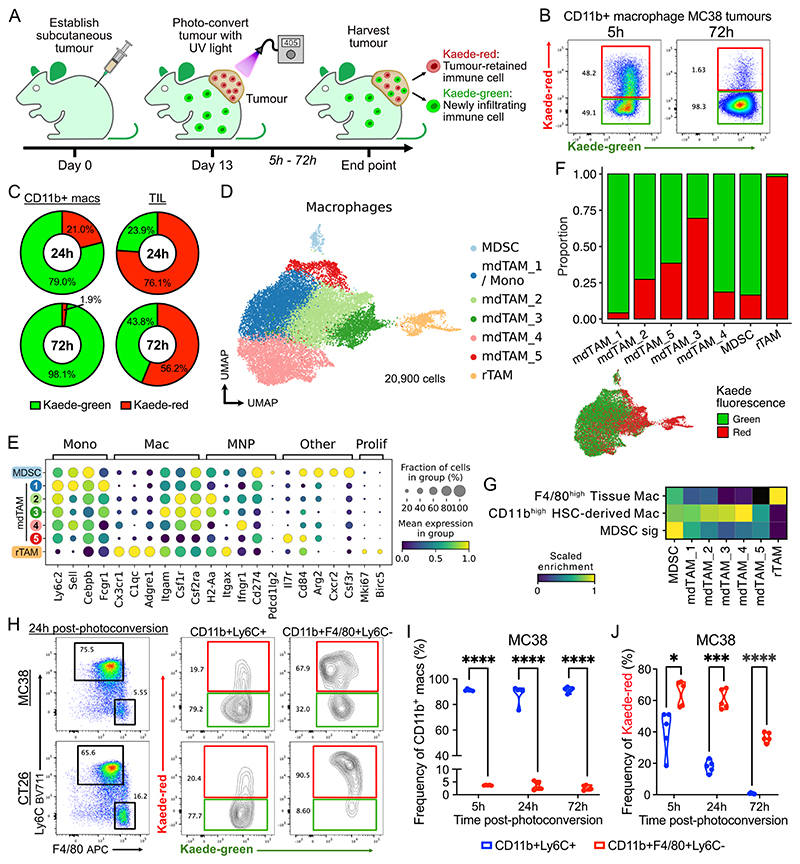
Temporal properties of tumour macrophages (A) Experiment set-up. (B) Representative flow cytometry of Kaede fluorescence of lineage^-^CD11b^+^ cells 5h and 72h post-photoconversion from subcutaneous MC38 tumours. (C) Flow cytometry quantification of Kaede fluorescence of lineage^-^CD11b^+^ cells versus tumour infiltrating lymphocytes (TIL; CD4 T, CD8 T, regulatory T, NK cells) post-photoconversion in MC38 tumours. Frequencies shown are mean of *n* >5 tumours per group. (D) Uniform Manifold Approximation and Projection (UMAP) of macrophages from scRNA-seq of TER119^-^CD45^+^Kaede^+^ cells, coloured by Leiden clusters. (E) Dot plot showing expression of selected marker genes in macrophage clusters. (F) Proportion of Kaede-green/red cells in macrophage clusters and UMAP of scRNA-seq of macrophages coloured by Kaede fluorescence profile. (G) Enrichment of published lineage-specific gene signatures (M-EXP-3510, GSE139125). (H) Representative flow cytometry showing Kaede fluorescence in macrophage subsets from MC38 and CT26 tumours 24h post-photoconversion. (I) Flow cytometry quantification of frequency of TAM subsets and (J) their Kaede fluorescence from MC38 tumours 5h to 72h post-photoconversion. Paired t-test with FDR correction was used; points represent tumours from independent mice (*n* = 5).

To enable site-specific temporal labelling of tumour-infiltrating leukocytes, we established syngeneic subcutaneous colorectal tumours (MC38-Ova, MC38 and CT26) in photoconvertible Kaede transgenic mice. Inoculated tumours were transcutaneously photo-labelled on day 13 by violet light, which converts all host cells within the tumour from the default green fluorescence (Kaede-green) protein to a red fluorescent profile (Kaede-red) with effectively 100% efficiency, due to optimised methodology (Supplementary Fig S1A) (25). Tumours were harvested between 5 and 72 hours following photoconversion and leukocytes were distinguished based on their Kaede fluorescence profile. Therefore, any Kaede-green^+^ cells represent leukocytes recruited since labelling, and Kaede-red^+^ cells indicate time-stamped leukocytes that have resided in the tumour since photoconversion (Fig 1A, Supplementary Fig S1B).

Photo-labelling revealed that the tumour myeloid compartment was highly dynamic, with rapid recruitment of CD11b^+^ cells during this stage of tumour growth. To our surprise, within 5h of photoconversion, only half of CD11b^+^ cells remained Kaede-red, and by 72h post-photoconversion, the tumour myeloid compartment predominantly consisted of newly-infiltrated Kaede-green cells in both tumour models (Fig 1B, Supplementary Fig S1C-E). The rate of change of CD11b^+^ cells was much greater than that of TILs, where approximately half remained Kaede-red up to 72h, as previously described (25) (Fig 1C, Supplementary Fig S1F).

To gain a broad overview of how myeloid cell states changed with tumour dwell, we performed bulk RNA-seq on sorted Kaede-green/red CD11b^+^ cells from tumours harvested 24h and 72h post-photoconversion (Supplementary Fig S1G). Tumour dwell-time (Kaede fluorescence), accounted for most of the transcriptional variance (Supplementary Fig S1H). Markers of macrophage residency, including *Cx3cr1* and *Adgre1* (which encodes F4/80), were more highly expressed in the Kaede-red fraction, while monocyte transcripts, such as *Ly6c2* and *Cebpb* were higher in the newly-infiltrated Kaede-green cells, as we expected (Supplementary Fig S1I). There was a tolerogenic shift in the transcriptional profile of CD11b^+^ cells, with *Il1b* and *Il6* transcripts more highly expressed in Kaede-green cells, and genes associated with an immunosuppressive state, including *Il10, Tgfb1*, and *Mrc1* higher in Kaede-red cells resident in the tumour for 24 to 72 hours. GSEA demonstrated that ‘*Hallmark reactive oxygen species*’, ‘*Hallmark TNFα signaling via NFκB*’ and ‘*Hallmark interferon γ response*’ genes were enriched in Kaede-green cells, while ‘*KEGG antigen processing and presentation*’ genes were enriched in Kaede-red cells (Supplementary Fig S1J). When comparing newly infiltrating Kaede-green cells present in the tumour for 24h with those present for 72h, we observed a change in expression of two transcription factors (TFs) critical for macrophage state, with down-regulation of *Cebpb* and upregulation of *Pparg* (Supplementary Fig S1I); the latter is associated with polarisation towards anti-inflammatory macrophages or tissue residency (37,38). Altogether, these data support the conclusion that rapid changes occur in the myeloid cell compartment, within hours to days of tumour entry, which our model enables us to track precisely.

### scRNA-seq identifies temporally distinct tumour macrophage populations

CD11b is expressed on a number of cell types, therefore, to better resolve the transcriptional changes occurring upon tumour entry we performed scRNA-seq of tumour-infiltrating CD11b^+^ myeloid cells, sorted on Kaede-green and Kaede-red fluorescence, 48h post-tumour photoconversion (Supplementary Fig S1G). After filtering for high-quality droplets, 31,931 single cells were analysed, of which 20,900 cells annotated as monocytes or macrophages based on canonical marker gene expression (Fig 1D, Supplementary Fig S2A-B).

Among macrophages, we annotated 5 clusters monocytes/monocyte-derived TAMs (mdTAM) because they included Kaede-green cells (Fig 1D-F), proving that they arose from circulating precursors. Indeed, mdTAMs expressed canonical monocyte genes, including *Ly6c2, Sell*, and *Cebpb*. These transcripts were most highly expressed in the mdTAM_1 cluster, which was predominantly (96%) Kaede-green and lacked *Cx3cr1* expression (Supplementary Fig S2C). This is consistent with their identity as newly infiltrating classical monocytes; hence, we labelled this cluster “mdTAM_1/Mono” (hereafter just “mdTAM_1”). One cluster was segregated from mdTAMs in the UMAP embedding and was annotated resident-like TAMs (rTAM), because 98% of these cells were Kaede-red^+^ (Fig 1F). In addition, rTAMs, but not mdTAMs, were enriched in cell-cycle genes, including *Mki67* and *Birc5* (Fig 1E, Supplementary Fig S2D), suggesting a capacity for self-maintenance *in situ*, a characteristic of prenatally-seeded tissue macrophages (17,18). Finally, one cluster was annotated myeloid-derived suppressor cells (MDSC), although it is important to note that there is ambiguity surrounding the definition of this cellular subset (39).

*Itgam* (CD11b) was expressed in both mdTAM and rTAM, but *Adgre1, Cx3cr1*, and *C1qc* were higher in rTAMs (Fig 1E). rTAMs also showed enrichment of reference transcriptional signature of fate-mapped prenatally-seeded tissue-resident F4/80^high^ macrophages (40) while mdTAMs were enriched for the HSC-derived F4/80^low^CD11b^+^ macrophage signature (Fig 1G).

To validate these observations, we performed flow cytometry on tumour macrophages and identified two distinct populations – a Ly6C-expressing F4/80^low-int^ (Ly6C^+^) population and a F4/80^high^ population lacking surface Ly6C (F4/80^+^Ly6C^–^), phenotypically resembling mdTAM and rTAM respectively (Fig 1H, Supplementary Fig S2E). Consistent with the scRNA-seq data, Ly6C^+^ cells were the dominant population, which include a spectrum of monocytes to monocyte-derived macrophages. Ly6C^+^ cells comprise >80% of TAMs, with their frequency remaining stable over time (Fig 1I, Supplementary Fig S2F). Ly6C^+^ mdTAMs were predominantly Kaede-green, compared to F4/80^+^Ly6C^–^ rTAMs, which were predominantly Kaede-red (Fig 1H), as observed in the scRNA-seq data. Ly6C^+^ TAMs showed near-complete replacement by Kaede-green immigrants by 72h in both MC38 and CT26 tumours, consistent with the conclusion that they are continuously replenished by circulating monocytes (Fig 1J, Supplementary Fig S2G). In contrast, a significantly greater proportion of F4/80^+^Ly6C^–^ rTAMs remained Kaede-red at 72h post-photoconversion, although this varied between MC38 and CT26 tumours. Together, these data support the presence of two phenotypically distinct TAM subsets with differing dynamics – mdTAMs that are constantly replaced by circulating precursors and an rTAM population with features of tumour residency.

### rTAM interact with fibroblasts at the tumour–normal interface

**Figure 2:**
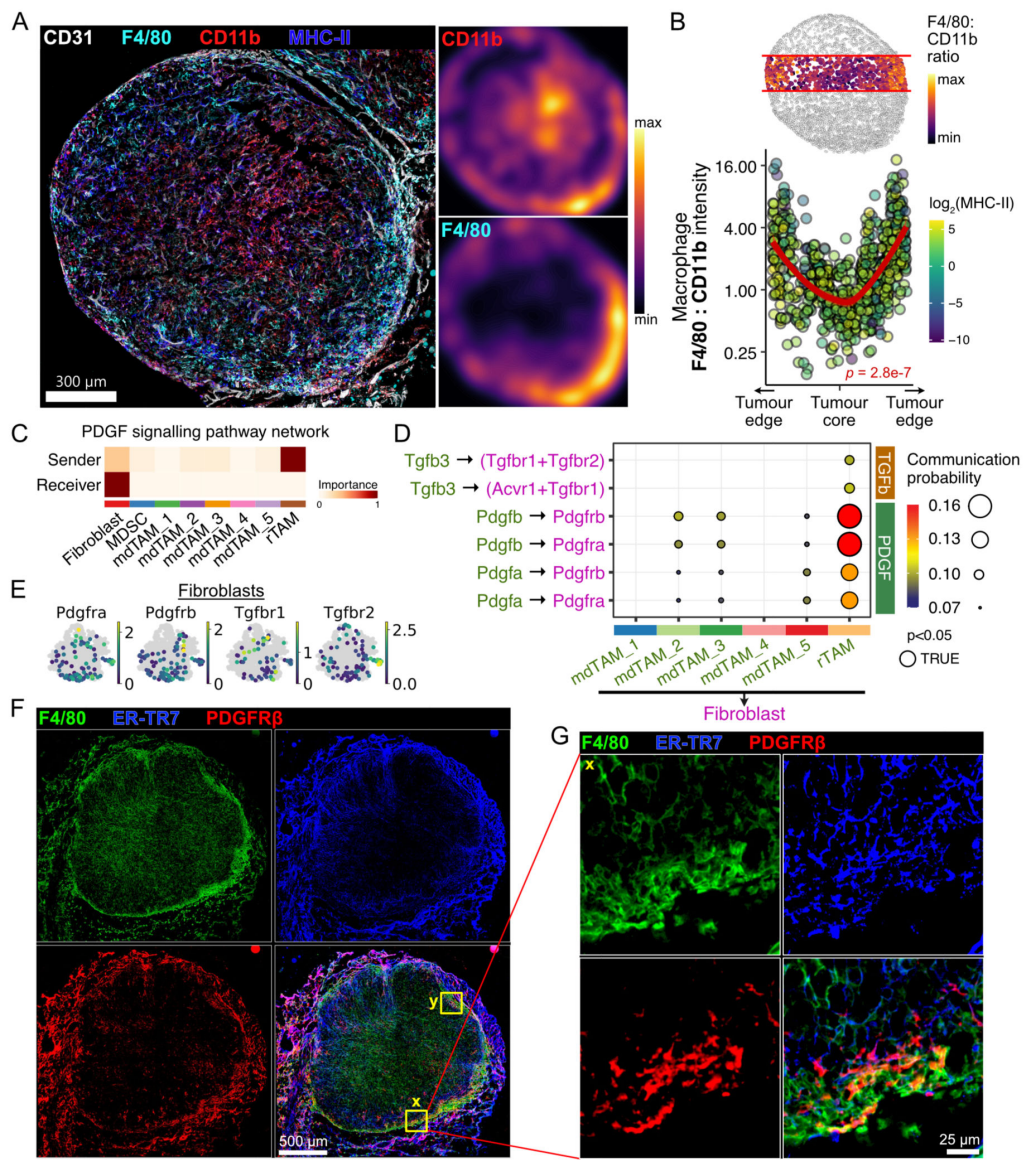
rTAM interact with fibroblasts at the tumour-normal interface (A) Immunofluorescence (IF) microscopy of CD11b^+^F4/80^+^ macrophages in subcutaneous MC38 tumours and kernel density heatmaps for staining of selected markers. (B) Quantification of F4/80-to-CD11b expression ratio in microscopy of tumour macrophages by location. *P-*value from Spearman correlation. (C) Cell-cell communication pathway analysis of scRNA-seq of tumour macrophages and fibroblasts. (D) Ligand-receptor interactions between TAMs and fibroblasts. (E) Expression of PDGF receptor and TGFβ receptor genes in scRNA-seq of stromal cells (fibroblasts). (F) IF microscopy of F4/80, ER-TR7 (fibroblasts/stroma), PDGFRβ in MC38 tumours. (G) High-resolution section of inset labelled ‘**x**’ from (E). Refer to Fig S3G for inset ‘**y**’. Imaging is representative of 3 independent experiments.

We next examined the spatial distribution of TAM subsets. We found that F4/80^high^ macrophages were preferentially located near the tumour–normal interface, whereas CD11b^+^F4/80^dim^ cells were more uniformly distributed, including within the tumour core (Fig 2A, Supplementary Fig S2H-I). CD11b was also expressed by neutrophils, which were present in tumours (Fig S1C); hence, we confirmed that most of the CD11b^+^ cells at the tumour core were macrophages rather than MPO^+^ neutrophils (Supplementary Fig S2J-K). Macrophage F4/80:CD11b expression ratio was highest at the tumour boundaries, and lowest at the centre (Fig 2B). Therefore, we conclude that temporally distinct mdTAM and rTAM subsets are localised to specific regions of the tumour.

Since F4/80^high^ rTAMs were spatially enriched at the tumour–normal interface, where a fibrous capsule forms surrounding subcutaneous tumours (Supplementary Fig S3A-B), we hypothesised that rTAMs may support fibroblasts and the formation of the tumour capsule. Cell–cell communication analysis identified prominent platelet-derived growth factor (PDGF) signalling between rTAM and *Vim^+^Fn1^+^Col6a1^+^* fibroblasts, but not other TAM subsets (Fig 2C, Supplementary Fig S3C). Indeed, ligand–receptor interactions mediated by *Pdgfa, Pdgfb*, and *Tgfb3*, which have well-established roles in fibroblast activation and survival (41), were specifically upregulated in rTAM (Fig 2D-E, Supplementary Fig S3D). We also found upregulation on rTAM of *Timp2*, which encodes a natural inhibitor of extracellular-matrix (ECM)-degrading proteases and may contribute to maintenance of the capsule (Supplementary Fig S3D). rTAM also expressed distinct integrin transcripts, including *Itga6, Itgb1*, and *Itgb5*, which bind ECM components including laminin and collagen, and may facilitate interaction at the tumour-normal interface (Supplementary Fig S3E). Consistent with the scRNA-seq cell–cell interaction analyses, a ring-like organisation of co-localised F4/80^high^ rTAMs and PDGF-receptor β^+^ fibroblasts was evident at the tumour–normal interface (Fig 2F-G, Supplementary Fig S3F-H). Altogether, these data suggest that rTAMs at the tumour–normal interface support fibroblasts via PDGF to coordinate ECM deposition.

### mdTAMs progress down one of two divergent trajectories defined by MHC-II following tumour infiltration

**Figure 3:**
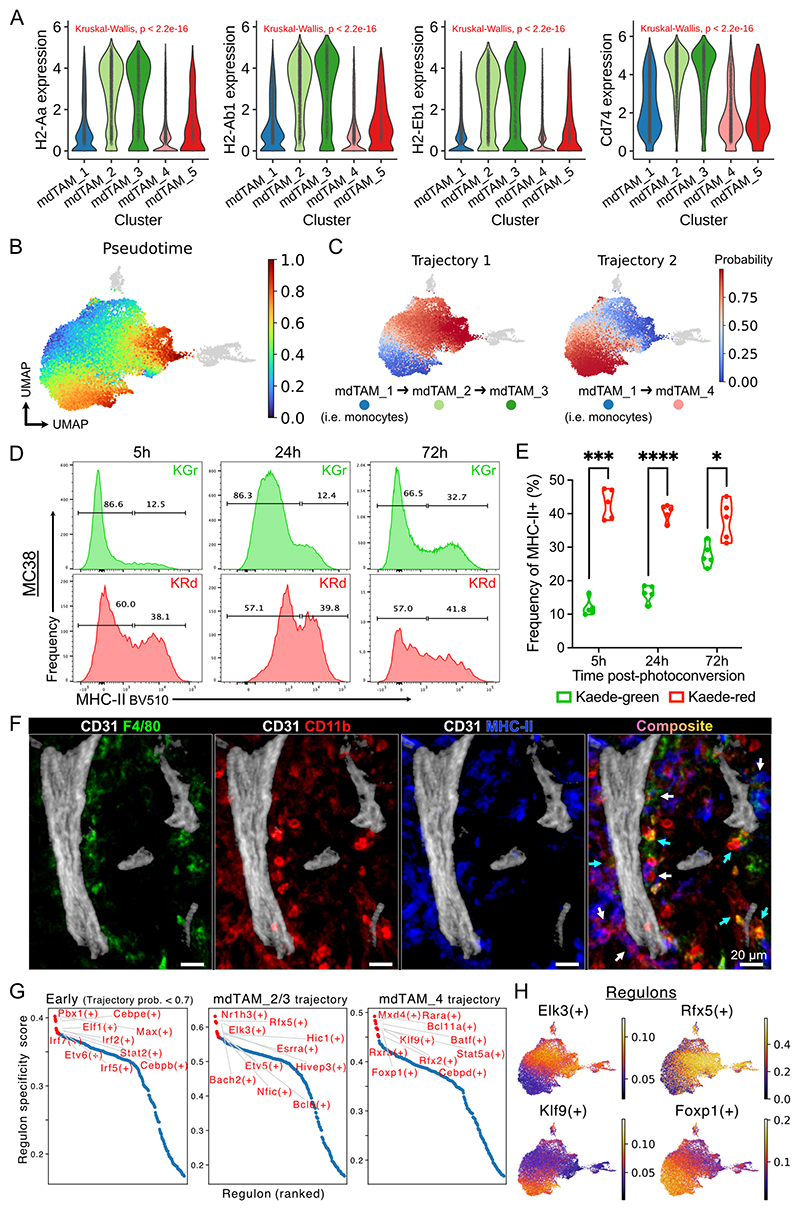
Monocytes entering the tumour differentiate following 2 trajectories defined by class II MHC (A) Expression of class II MHC (MHC-II) genes in scRNA-seq data of mdTAM clusters from MC38 tumours. Kruskal-Wallis test was used. (B-C) Palantir pseudotime analysis demonstrating two independent maturation trajectories in mdTAMs. Colour in (B) represents pseudotime values from 0 (earliest) to 1 (latest), and (C) represents probability of a cell belonging to the specified trajectory. (D) Representative flow cytometry of surface MHC-II expression on lineage^-^CD11b^+^Ly6C^+^ mdTAMs, by Kaede fluorescence, 5h to 72h post-photoconversion in MC38 tumours. (E) Quantification of (D). Paired t-test with FDR correction was used; points represent tumours from independent mice (*n* = 5). (F) IF microscopy of CD11b^+^F4/80^+/-^MHC-II^+^ macrophages (white arrows) and CD11b^+^F4/80^+/-^ MHC-II^-^ macrophages (cyan arrows) with CD31^+^ blood vessels in MC38 tumours. Representative of 3 independent experiments. (G) Gene regulatory network transcription factor (TF) inference analysis; regulon specificity score-ranked TFs by mdTAM trajectory, with top 10 regulons labelled. (H) Regulon activity score for selected differentially enriched regulons between mdTAM trajectories.

In contrast to rTAMs, the abundant, rapidly replenished mdTAMs were present throughout the tumour (Fig 2). In homeostasis, a divergent fate of incoming monocytes towards the development of class-II MHC (MHC-II)^+^ or MHC-II^–^ macrophage populations has been described in multiple tissues, including lungs and kidney (42,43). Similarly, MHC-II^+^ and MHC-II^–^ macrophages have been identified in tumours (20,24,44), but their developmental relationship and the timing of their emergence following monocyte entry into tumours remain unclear. Analysis of the single-cell transcriptomes of mdTAMs showed transcriptional heterogeneity, with the mdTAM_2/3 clusters expressing significantly higher levels of MHC-II transcripts and the MHC-II invariant chain *Cd74* compared with the newly infiltrating monocytes (i.e. mdTAM_1) and mdTAM_4/5 (Fig 3A).

An entropy-based pseudotime analysis rooted in the mdTAM_1/monocyte cluster (because it comprised of 96% Kaede-green cells) revealed two trajectories within mdTAMs: trajectory 1 terminating in mdTAM_3 via an mdTAM_2 intermediate, and trajectory 2 terminating in mdTAM_4 (Fig 3B-C). This binary fate was confirmed by diffusion mapping, which also showed mdTAMs situated along two major branches (Supplementary Fig S4A-B). In addition, we applied partition-based graph abstraction (PAGA), which validated similar connectivity between clusters (Supplementary Fig S4C). Moreover, MHC-II transcripts were the top differentially expressed genes (DEGs) enriched along the mdTAM_2/3 trajectory (Supplementary Fig S4D-E). Reference gene signatures (42) derived from homeostatic MHC-II^high^ and MHC-II^low^ monocyte-derived macrophages were enriched in mdTAM_2/3 and mdTAM_4, respectively (Supplementary Fig S4F), further supporting the conclusion that tumour-infiltrating monocytes progress down one of two developmental trajectories.

To assess how rapidly this phenotypic heterogeneity developed in infiltrating monocytes, we investigated mdTAM populations longitudinally post-photoconversion using flow cytometry. Among newly infiltrated Kaede-green CD11b^+^Ly6C^+^ cells in MC38 tumours, there were few MHC-II^+^ cells 5h post-photoconversion, but this gradually increased over time, such that nearly 30% expressed MHC-II at 72 hours (Fig 3D-E). In Kaede-red CD11b^+^Ly6C^+^ cells present in the tumour since photo-labelling, ∼40% expressed MHC-II, and this remained stable over time. Hence, these data support that new tumour-infiltrating monocytes rapidly (within a matter of hours) differentiate into MHC-II^+^ TAMs or remain MHC-II^–^. These changes were confirmed in CT26 tumours, but the dynamics and output of mdTAM differentiation differed from MC38 tumours, with 30% of Kaede-green CD11b^+^Ly6C^+^ cells showing MHC-II expression by 24 hours, and ultimately ∼65% of Kaede-red mdTAMs were MHC-II^+^ (Supplementary Fig S4G). This suggests that tumour-intrinsic factors can influence the fate decision of incoming monocytes.

In homeostasis, the two monocyte-derived macrophage subsets were previously described to occupy different spatial locations, with MHC-II^–^ macrophages lying adjacent to blood vessels and MHC-II^+^ macrophages in close proximity to nerves (42). We observed bimodal expression of MHC-II on CD11b^+^MPO^–^ macrophages on imaging of tumour sections (Supplementary Fig S4H). However, MHC-II^+/–^ mdTAMs enriched within the core of the tumour were intermingled, with no obvious predilection of either subset for blood vessels (Fig 3F, Supplementary Fig S4I), and no nerves detectable within subcutaneous tumours (Supplementary Fig S4J).

Finally, we investigated differentially enriched TF regulon activity between TAM clusters, since we hypothesized that their distinct spatiotemporal features and diverging trajectories were likely driven by cell-type/state-specific gene regulatory networks. mdTAMs were enriched for monocyte-associated TF activity, such as *Cebpb* and *Irf7* (Supplementary Fig S4K), whereas *Pparg*, which has been associated with resident macrophages in multiple tissues (37), and *Myc* activity, consistent with a proliferative state, were increased in rTAM (Supplementary Fig S2D). Among mdTAMs, ranked regulon specificity scores showed distinct TF regulons enriched with mdTAM_2/3 or mdTAM_4 trajectories, but there were shared monocyte-associated regulons earlier in pseudotime (Fig 3G). We observed that *Elk3* and *Rfx5* regulons were specifically enriched in the mdTAM_2/3 trajectory, and these are known to facilitate macrophage phagocytosis and MHC-II expression (45,46) (Fig 3H). Meanwhile, TFs involved in cytokine production or regulation of inflammation, such as *Klf9* or *Foxp1* (47), were higher in mdTAM_4. Of note, previous work has associated MHC-II^+^ and MHC-II^–^ TAMs with “M1”- and “M2”- polarised macrophages respectively, or suggested that MHC-II^–^ TAMs possess immunosuppressive properties (4,6,44). However, we did not observe a predilection for RNA expression of “M1”- and “M2”- associated genes in any specific TAM cluster, reinforcing that this paradigm is over-simplified (3,9), nor increased expression of immunosuppressive molecules in MHC-II^–^ mdTAMs (Supplementary Fig S5A-B).

### MHC-II^+^ and MHC-II^–^ mdTAM are functionally distinct

**Figure 4:**
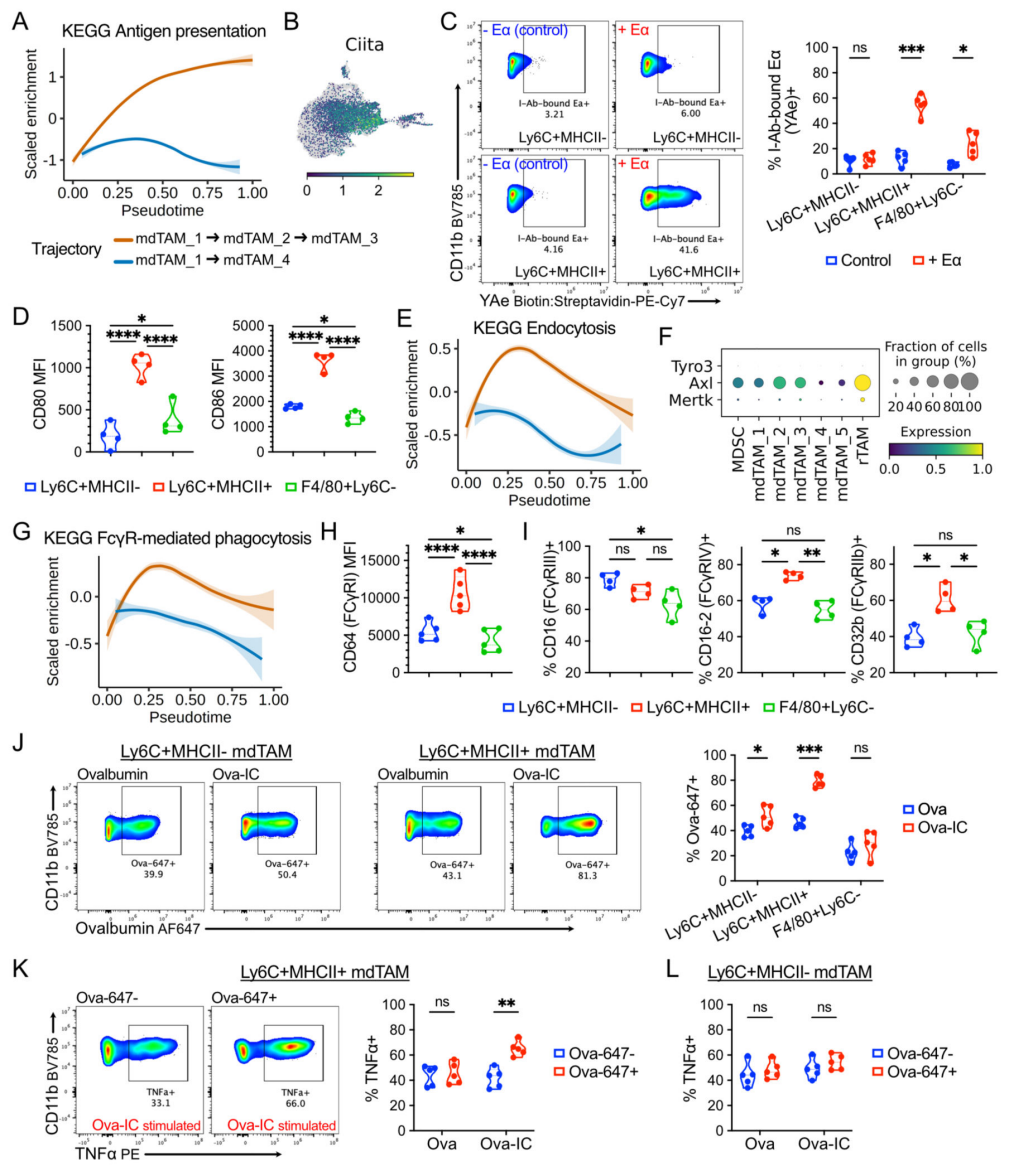
Functionally distinct monocyte-derived TAM populations (A) Enrichment scores for ‘*KEGG antigen processing and presentation*’ across pseudotime in mdTAM trajectories. (B) Expression of *Ciita*. (C) Flow cytometry of Eα peptide bound to I-Ab (MHC-II) across TAM clusters in *ex vivo* tumour cultures; representative plots and quantification. (D) Flow cytometry of surface CD80 and CD86 expression in MC38 tumours. (E) Enrichment scores for ‘KEGG endocytosis’ across pseudotime. (F) Expression of ‘TAM’ family receptor tyrosine kinase genes. (G) Enrichment scores for ‘KEGG FcγR-mediated phagocytosis’ across pseudotime. (H) Flow cytometry of surface FcγRI expression in MC38 tumours, and (I) surface FcγRIII, FcγRIV and FcγRIIb expression. (I) Flow cytometry of fluorescent AF647-conjugated ovalbumin (Ova-647)+ cells in Ova-647 versus Ova-647- immune complex treated tumours *ex vivo*; representative plots and quantification. (J) Flow cytometry of TNFα expression in Ova-647+ Ly6C^+^MHC-II^+^ mdTAM (i.e. cells that have bound or internalised Ova-647/Ova-647-immune complexes) versus Ova-647-cells, and representative flow plots; and the same comparison in Ly6C^+^MHC-II^-^ mdTAM (L). One-way ANOVA (paired data) with Šidák’s multiple comparisons test was used (D,H-I); paired t-tests with FDR correction were used (C,J-L). Points represent tumours from independent mice (C,H,J,K,L: *n* = 5; D,I: *n* = 4).

To explore whether the mdTAM populations that emerged following monocyte infiltration into the tumour were functionally distinct, we first assessed the expression of genes involved in antigen processing and presentation beyond MHC-II. Tapasins (*Tap1/2*), proteasomal units (*Psme1/2*), cathepsins (*Ctss*), chaperones (*B2m, Tapbp*), and *Ciita*, a master regulator of MHC antigen presentation (48) were all upregulated along the mdTAM_2/3 trajectory, in contrast to the mdTAM_4 trajectory (Fig 4A-B, Supplementary Fig S5C). To confirm whether these transcriptional changes actually led to an enhanced ability of Ly6C^+^MHC-II^+^ mdTAM to process and present antigen, we incubated tumour single-cell suspensions *ex vivo* with Eα(52–68) peptide for 2h. Ly6C^+^MHC-II^+^ but not Ly6C^+^MHC-II^–^ mdTAM exhibited increased presentation of Eα on surface I-Ab MHC-II (Fig 4C). F4/80^+^Ly6C^–^ rTAM, which express MHC-II, could also present antigen, but were less efficient than Ly6C^+^MHC-II^+^ mdTAM (Fig 4C). Moreover, Ly6C^+^MHC-II^+^ mdTAM demonstrated higher CD80 and CD86 expression, potentially suggesting an increased co-stimulatory capacity (Fig 4D).

Beyond MHC-II expression and antigen presentation, we also found enrichment of an “endocytosis” geneset in the mdTAM_2/3 trajectory compared with the mdTAM_4 trajectory (Fig 4E). Indeed, *Axl*, a receptor known to be involved in the recognition and uptake of apoptotic tumour cells (49), was upregulated in mdTAM_2/3 (Fig 4F). Consistent with a greater capacity for tumour antigen internalisation, there was also enrichment of an “FcγR-mediated phagocytosis” geneset in the mdTAM_2/3 trajectory (Fig 4G). This was of particular interest given the increasing appreciation of the potential for tumour-coating IgG to facilitate anticancer responses (50), including in an FcyR-dependent manner (51). Expression of Fcγ-receptor (FcγR)I, a high affinity receptor that binds monomeric IgG (52), was higher in MHC-II^+^ mdTAM_2/3 among TAM subsets (Fig 4H, Supplementary Fig S5D). In addition, the expression of low affinity activating (FcγRIII/FcγRIV) and inhibitory (FcγRIIb) FcγRs, which collectively regulate cellular activation by IgG immune complexes (52), was higher in Ly6C^+^MHC-II^+^ mdTAM_2/3 compared to Ly6C^+^MHC-II^–^ mdTAM_4 and F4/80^+^Ly6C^–^ rTAMs (Fig 4I, Supplementary Fig S5E), suggesting a greater capacity for immune complex–mediated activation.

We therefore hypothesised that MHC-II^+^ mdTAM preferentially bind and uptake IgG immune complexed tumour antigen, potentially augmenting antitumour immunity. To test this, we administered AF647-labelled fluorescent Ova (Ova-647) or Ova-IC to tumours *ex vivo*. At 6 hours, Ly6C^+^MHC-II^+^ mdTAM had internalised Ova-IC more effectively than other TAM subsets (Fig 4J). Furthermore, in Ova-IC–stimulated tumours, we observed increased intracellular TNFα in Ly6C^+^MHC-II^+^ mdTAMs that had bound or internalised Ova-IC compared to Ova-647-stimulated cells (Fig 4K), but this increase was not present in Ly6C^+^MHC-II^–^ mdTAMs (Fig 4L).

Altogether, these data support the conclusion that monocytes entering the tumour differentiate in a binary manner into one of two functionally distinct mdTAM subsets/states, distinguished by MHC-II expression, with MHC-II^+^ mdTAMs showing a greater capacity to internalise and respond to IgG-opsonised tumour antigen, and for immunogenic antigen presentation.

### Genetically modified pre-clinical models of colon cancers have analogous TAM populations

Next, we sought to assess whether similar macrophage populations are evident within preclinical models that more closely mimic the natural progression of spontaneous colorectal cancers and would likely be of greater physiological relevance (53–55). We analysed scRNA-seq data of tumour-infiltrating monocytes and macrophages from orthotopically transplanted *Kras*^G12D/+^*Trp53*^fl/fl^*Notch1*^Tg^ (KPN) and *Apc*^fl/fl^*Kras*^G12D/+^*Trp53*^fl/fl^*Tgfbr1*^fl/fl^ (AKPT) tumour organoids, as well as tamoxifen-induced *Villin-cre*^ERT2^ *Apc*^fl/fl^ colon tumours (Research Square preprint doi.org/10.21203/rs.3.rs-6812365/v1). Analogous TAM states with high transcriptional similarity were identified across all models (Supplementary Fig S6). We observed that population frequencies differed by model, for example, *Apc*^fl/fl^ tumours demonstrated relatively higher abundance of rTAMs (Supplementary Fig S6E). Hence, these data support the translatability of our findings and indicate that TAM states with differing functional properties vary in frequency in tumours with different genetic backgrounds.

### Anti-PD-L1 treatment attenuates rapid loss of pro-inflammatory gene expression following tumour infiltration

**Figure 5:**
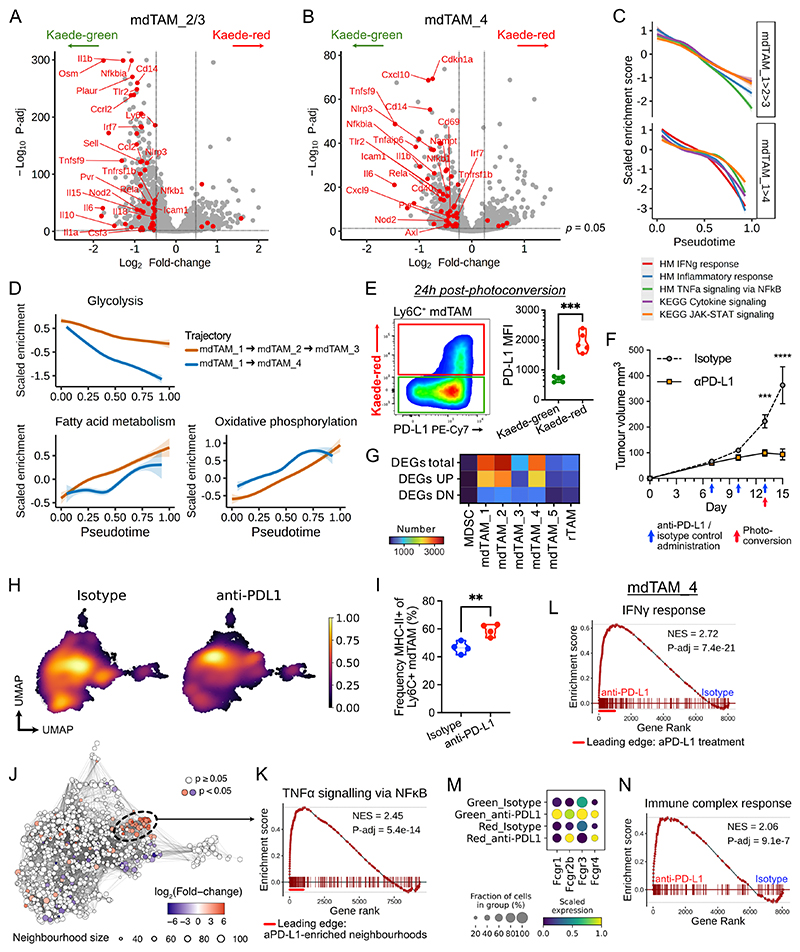
Rapid changes in gene expression following TAM infiltration attenuated by anti-PD-L1 treatment (A-B) Differential gene expression between Kaede-red and Kaede-green cells in mdTAM_2/3 (trajectory 1, A) and mdTAM_4 (trajectory 2, B). Significant DEGs from the ‘*Hallmark inflammatory response*’ geneset are highlighted. (C) Enrichment scores for selected pro-inflammatory genesets across pseudotime in mdTAM trajectories. (D) Enrichment scores for Hallmark metabolic gene sets across pseudotime. (E) Flow cytometry of surface PD-L1 expression in Ly6C^+^ TAMs 24h post-photoconversion, from MC38 tumours. Paired t-test was used. (F) Tumour (MC38-Ova) growth curves comparing anti-PD-L1 antibody-treated versus isotype control antibody treatment, administered on day 7, 10 and 13. Data shown is related to the scRNA-seq experiment and includes 5 mice per condition. Two-way ANOVA and Sidak’s multiple comparisons test was used; the data is presented as means ± SEM. (G) Heatmap of number of DEGs in TAM clusters; anti-PD-L1 versus isotype control-treated tumours. (H) Gaussian kernel density in UMAP embedding of TAMs. (I) Frequency of Ly6C^+^MHC-II^+^ mdTAM following anti-PD-L1 treatment of MC38 tumours *in vivo*. Student’s t-tests with FDR correction were used; points represent tumours from independent mice (E: *n* = 5, I: *n* = 4). (J) Milo differential abundance analysis of TAMs in anti-PD-L1-treated versus isotype control-treated tumours. Points represent overlapping cellular neighbourhoods. Dotted ellipse highlights a cluster of neighbourhoods enriched in anti-PD-L1-treatment. (K) GSEA of selected neighbourhoods encircled in (J) versus non-anti-PD-L1 enriched Kaede-red majority (>60%) neighbourhoods in mdTAM_2/3. (L) GSEA of ‘Hallmark IFNγ response’ in mdTAM_4, comparing anti-PD-L1 versus isotype control-treated tumours. (M) Expression of FcγR transcripts in mdTAM_4. (N) GSEA of an immune complex response signature (GSE200033) in mdTAM_4.

Although mdTAMs undergo divergent maturation programmes following tumour influx (Fig 3), both mdTAM trajectories showed a similar loss of inflammatory gene expression with increasing tumour dwell-time. Transcripts associated with acute inflammation (eg. *Tnfrsf1b, Nlrp3, Nfkb1*), lymphocyte recruitment and activation (*Ccl2, Cxcl9, Tnfsf9*), and cytokines (*Il1b, Il6, Il15*) were downregulated in Kaede-red mdTAMs compared with their Kaede-green counterparts (Fig 5A-B). GSEA revealed that multiple pathways involved in immune activation were suppressed in Kaede-red mdTAMs, including “*Interferon gamma response*”, “*Inflammatory response*” and “*Cytokine-cytokine receptor interaction*” (Supplementary Fig S7A-B). Moreover, the anti-inflammatory transcriptional changes induced by the tumour environment were evident by 48h post-photoconversion.

To precisely order time-associated changes following tumour influx, we plotted enrichment scores over pseudotime, which emphasised the rapid downregulation of immunogenic transcripts following tumour entry (Fig 5C, Supplementary Fig S7C). Among the leading-edge genes for these genesets were pro-inflammatory cytokines and co-stimulatory molecules, such as *Tnf, Il1b, Cd80* and *Cd86* (Supplementary Fig S7D). TF regulatory networks critically involved in macrophage inflammation, including NFκB (8), and *Irf1, Stat1* and *Stat2* regulons downstream of interferon activation were also concomitantly downregulated in both trajectories (Supplementary Fig S7E). Moreover, *glycolysis* genesets, classically associated with a pro-inflammatory state, decreased following tumour influx, while *oxidative phosphorylation* and *fatty acid metabolism*, associated with anti-inflammatory states, was increased (Fig 5D). We therefore examined surface expression of CD206 and PD-L1, markers of tolerogenic macrophages (4,15). Both were significantly higher on Kaede-red compared to Kaede-green mdTAMs as early as 24h after photo-conversion (Fig 5E, Supplementary Fig S7F-G), highlighting that substantial changes to the mdTAM phenotype are quickly induced following tumour entry.

Given the high expression of PD-L1 on tumour-retained macrophages, we next sought to understand how ICB using anti-PD-L1 might influence the transcriptional state of the cells. Immune checkpoint inhibitors have become the standard-of-care in advanced malignancies and disrupt suppressive myeloid–T cell interactions (12). Treatment with anti-PD-L1 significantly restricted MC38-Ova tumour growth *in vivo* (Fig 5F), and had a variable effect on different TAM populations. The total number of DEGs between anti-PD-L1-treated and isotype control-treated tumours was markedly lower in rTAM compared to mdTAM (Fig 5G), despite similar expression of PD-L1 (Supplementary Fig S8A). Within mdTAM, monocytes (i.e. mdTAM_1), mdTAM_2 and 4 had the greatest response to anti-PD-L1 treatment in terms of number of DEGs (Fig 5G). These populations are predominantly Kaede-green (Fig 1F), suggesting that newly-infiltrated mdTAMs are the primary responders to anti-PD-L1, and mdTAMs are less amenable to activation by anti-PD-L1 with more prolonged tumour dwell time. GSEA showed many pathways were similarly enriched across mdTAM_2/3, mdTAM_4 and rTAM in anti-PD-L1-treated tumours, including ‘*TNFα signaling via NFκB*’ and ‘*Inflammatory response*’ (Supplementary Fig S8B). In addition, the *Nfkb1* regulon, a central feature of macrophage activation (8), was upregulated in both mdTAM lineages (Supplementary Fig S8C). Together, these data support the conclusion that anti-PD-L1 treatment results in pan-TAM activation, but that increasing tumour dwell-time renders macrophages more resistant to this activating stimulus.

Kernel density analysis of the TAM landscape revealed that in anti-PD-L1-treated tumours, mdTAMs were denser in UMAP embeddings corresponding to earlier points in pseudotime, particularly in the mdTAM_2/3 trajectory (Fig 5H). Flow cytometric assessment identified three factors underpinning this observation. First, there was a significant increase in the ratio of Ly6C^+^ mdTAM to F4/80^+^Ly6C^–^ rTAM in anti-PD-L1-treated tumours, indicating an increased influx of monocyte-derived macrophages (Supplementary Fig S8D). Second, there was an increased proportion of MHC-II^+^ cells among CD11b^+^Ly6C^+^ macrophages (corresponding to mdTAM_2/3 in our scRNA-seq data) (Fig 5I), suggesting a skewing of monocyte differentiation towards the MHC-II^+^ fate with anti-PD-L1 treatment. Last, there was an upregulation of surface CD80 and CD86 expression in the Ly6C^+^MHC-II^+^ macrophages (Supplementary Fig S8E), suggesting that anti-PDL-L1 therapy attenuated the loss of inflammatory gene expression following tumour infiltration. Of note, these changes in CD80/86 expression with anti-PDL1 treatment were not observed in Ly6C^+^MHC-II^–^ or F4/80^+^Ly6C^–^ cells, indicating subset-specific effects on TAMs. Consistent with the flow cytometric data, neighbourhood-based differential abundance analyses showed increases in mdTAM_2 neighbourhoods and a relative reduction from mdTAM_3 in the mdTAM_2/3 trajectory in anti-PD-L1-treated tumours (Fig 5J, Supplementary Fig S8F). The anti-PD-L1 enriched mdTAM_2 neighbourhoods were tightly clustered in the UMAP embedding and are predominantly Kaede-red (Fig 5J), indicating that these cells had been in the tumour for at least 48 hours. Compared to the remaining Kaede-red cell neighbourhoods in this trajectory, cells in the anti-PD-L1-associated neighbourhoods were specifically enriched for NFκB-regulated signalling, regulons, and inflammatory gene expression (Fig 5K, Supplementary Fig S8G-H). These data indicate that the downregulation of activation programmes in MHC-II^+^ mdTAMs following tumour entry is mitigated by anti-PD-L1 treatment.

We also found changes in mdTAM_4 in anti-PD-L1-treated tumours, with several inflammatory pathways preferentially enriched, particularly ‘*Hallmark Interferon gamma response*’ (Fig 5L, Supplementary Fig S8B,I). At baseline, mdTAM_4 was particularly high in *Ifngr1* expression, despite low expression of genes associated with IFNγ stimulation, potentially indicating an unutilised capacity to respond to type-II interferon that is held in check by the tumour environment (Supplementary Fig S8J). Following anti-PD-L1 treatment, there was evidence of increased IFNγ receptor-mediated activation, with increased *Stat1* and *Irf1* regulon activity (Supplementary Fig S8K). FcγR gene expression was also significantly upregulated on mdTAM_4 following treatment (Fig 5M), along with enrichment for a gene signature representing macrophage response to IgG-immune complexes (56) (Fig 5N). Hence, ICB may enhance the ability of MHC-II^–^ mdTAM_4 to respond to both IFNγ and IgG-opsonised tumour cells/antigens.

### Anti-PD-L1 promotes an IFNγ-driven positive feedback loop between mdTAMs and CD4^+^ T cells

**Figure 6:**
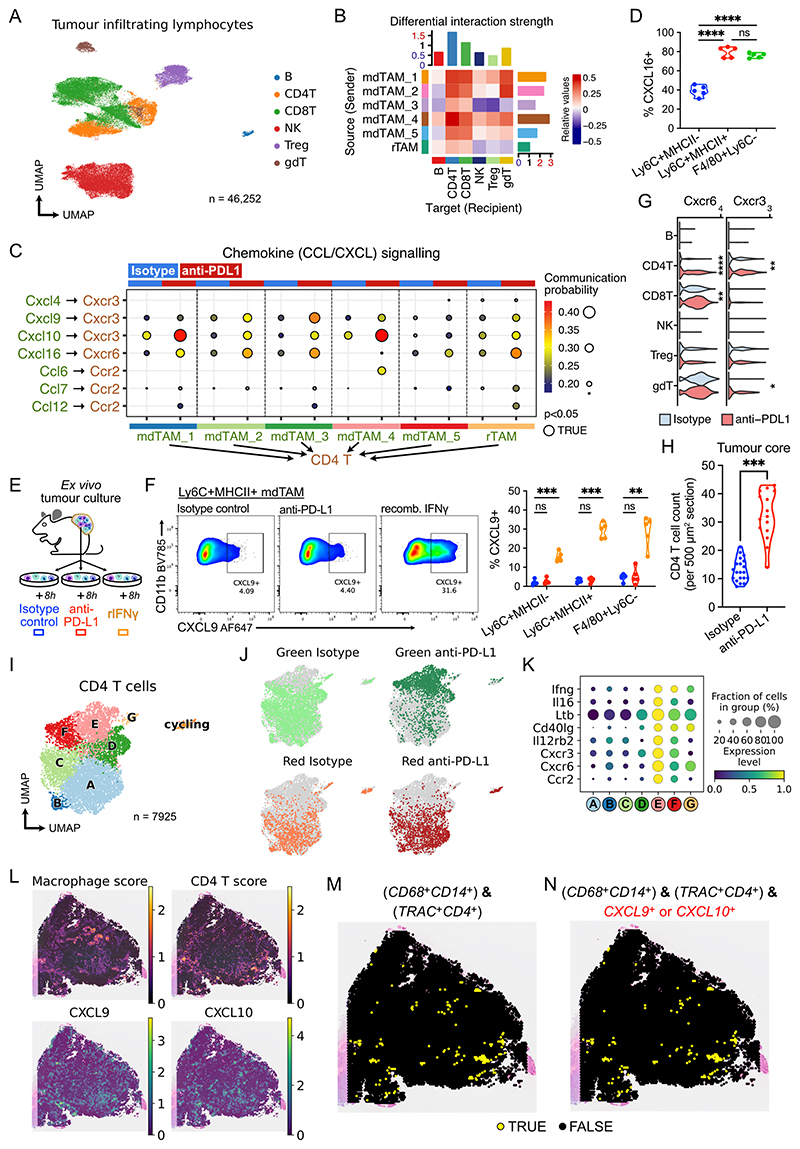
Anti-PD-L1 alters macrophage-lymphocyte interaction in the tumour, in a subset specific manner (A) UMAP of scRNA-seq of tumour-infiltrating lymphocytes (TIL), coloured by broad cell types. (B) Cell-cell communication analysis between TAMs (signal source) and TILs (signal targets), indicating differential interaction strength between cells from anti-PD-L1-treated (value>0) versus isotype control-treated (value<0) tumours. Bars represent mean values across sources (rows) or targets (columns). (C) Ligand-receptor interactions from chemokine signalling pathways between TAMs and CD4^+^ T cells. (D) Flow cytometry of intracellular CXCL16 expression in TAMs from unstimulated tumours. One-way ANOVA with Šidák’s multiple comparisons test was used. (E) Experiment set up for *ex vivo* culture. (F) Flow cytometry of intracellular CXCL9 expression in TAMs from tumours treated with isotype control, anti-PD-L1 antibodies, or recombinant IFNγ *ex vivo* for 8h; representative plots and quantification. Paired t-test with FDR correction was used. Points represent tumours from independent mice (D,F: *n* = 5). (G) *Cxcr3* and *Cxcr6* expression in scRNA-seq of TILs. (H) Quantification of tumour-infiltrating CD4^+^ T cells from IF microscopy of isotype control or anti-PD-L1-treated tumours *in vivo*. Student’s t-test was used; points represent sections from 3 independent experiments. (I) UMAP of CD4^+^ T cell subset from (A). (J) CD4^+^ T cells by Kaede-fluorescence and treatment group. (K) Selected gene expression in CD4^+^ T cell clusters. (L) Visium spatial transcriptomics of human colorectal tumour; macrophage and CD4^+^ T cell gene enrichment and CXCL9/10 expression. (M) Spots with expression of *CD68* and *CD14* (monocytes/macrophages), and *TRAC* and *CD4* (CD4^+^ T cells). (N) Spots fulfilling conditions in (M) and expressing *CXCL9* or *CXCL10*.

Given the increase in lymphocyte-targeting cytokine and chemokine transcripts in TAMs following anti-PD-L1 treatment (Supplementary Fig S8L), we integrated scRNA-seq data from TILs and performed cell–cell communication analysis to interrogate how TAM subset interactions with TILs were affected by anti-PD-L1 treatment (Fig 6A). First, to determine which TAM subset to TIL cell-type interactions were most affected by ICB, we compared differential interaction strengths in anti-PD-L1 versus isotype control-treated tumours (Fig 6B). Among TAMs, monocytes (i.e. mdTAM_1), and mdTAM_2 and 4 had the largest increase in outgoing (“sender”) signal strength following anti-PDL1 treatment, and there was little change from rTAMs. Overall, the largest effect of anti-PD-L1 treatment was on TAM interactions with CD4^+^ T cells (Fig 6B), with a >50% increase in weighted interaction strength between TAMs and CD4^+^ T cells (Supplementary Fig S9A). Comparatively, the weighted interaction strength with CD8^+^ T cells increased by only 8%.

Functional annotation of mdTAM-to-CD4^+^ T-cell signalling pathways showed a significant increase in CXCL-driven chemotactic signals in anti-PD-L1 treatment (Supplementary Fig S9B), most prominently *Cxcl9–Cxcr3, Cxcl10–Cxcr3* and *Cxcl16–-Cxcr6* (Fig 6C). TAM subset-specific differences in interactions were evident, with *Cxcl10–Cxcr3* signalling prominent in the mdTAM_1>4 trajectory and *Cxcl9–Cxcr3* signalling upregulated in the mdTAM_1>2>3 trajectory (Fig 6C). The *Cxcl16–Cxcr6* interactions were evident in rTAMs and in mdTAM_2/3 but not in mdTAM4 (Fig 6C). To validate this transcriptional data at the protein level, we assessed intracellular chemokine expression in the TAM subsets by flow cytometry. Consistent with our scRNA-seq analysis, at baseline, CXCL16 was highly expressed by rTAM and MHC-II^+^ mdTAM (∼80% CXCL16^+^), with much lower expression in MHC-II^**–**^ mdTAMs (Fig 6D, Supplementary Fig S9C-D). Across the different TAM populations, 2-5% were CXCL9^+^ at baseline, with less variation between subsets (Supplementary Fig S9E).

We next sought to investigate the mechanism by which anti-PD-L1 treatment might enhance TAM chemokine production. Given that TAMs express PD-L1, this might occur by direct ligation of PD-L1 in a cell-intrinsic manner, or due to a secondary signalling molecule produced by a non-macrophage population following anti-PD-L1 treatment. We reasoned that IFN_*γ*_ was a prime candidate stimulatory signal, since IFNγ response genes were induced in TAMs following anti-PD-L1-treatment (Supplementary Fig S8) and enhanced IFNγ signalling is a hallmark of effective ICB (57). To test these possibilities, we stimulated treatment-naïve tumours *ex vivo* with isotype control, anti-PD-L1 antibodies, or recombinant IFNγ (Fig 6E). Anti-PD-L1 treatment had no effect on TAM CXCL9 production in these isolated tumour cultures (Fig 6F). In contrast, IFNγ stimulation significantly increased TAM CXCL9 expression, with the greatest upregulation evident in Ly6C^+^MHC-II^+^ mdTAM, mirroring the effects on anti-PD-L1-treated tumours *in vivo*. Neither anti-PD-L1 nor IFNγ administration *ex vivo* affected CXCL16 expression (Supplementary Fig S9F), hence CXCL16 may be regulated by IFNγ-independent mechanisms. Moreover, these data suggest that anti-PD-L1-associated increases in TAM chemokines require *de novo* recruitment of circulating cells or the introduction of additional circulating factors, and cannot be mediated by cells already present in the tumour (i.e. *ex vivo* tumour preparations).

Given the increase in *Cxcl9/10* mediated signals from mdTAMs following anti-PD-L1 treatment, we hypothesised that *Cxcr3-*expressing T cells would localise to the tumour core, where mdTAMs were enriched (Fig 2). *Cxcr3* expression was relatively specific to CD4^+^ T cells and increased following anti-PD-L1 treatment (Fig 6G), and an increase in CD4^+^ T cells was evident in the tumour core in anti-PD-L1-treated tumours (Fig 6H, Supplementary Fig S9G). Further analysis of scRNA-seq of CD4^+^ T cells in isolation revealed two clusters (labelled “**E**” and “**F**”), that were predominantly newly recruited Kaede-green cells and almost exclusively present in anti-PD-L1-treated tumours (Fig 6I-J). These CD4T_**E**/**F** clusters showed the highest expression of *Cxcr3* and *Cxcr6*, but also *Ifng* (Fig 6K) and there was a reciprocal increase in CD4^+^ T cell-to-TAM IFNγ signalling following anti-PD-L1 treatment (Supplementary Fig S9H). Taken together with our observation that treatment of *ex vivo* tumours with IFN_*γ*_ led to an increase in CXCL9 (a CXCR3 ligand) in mdTAMs (Fig 6F), these data are consistent with the presence of a positive feedback loop where CXCL9 or CXCL10 derived from MHC-II^+^ or MHC-II^–^ mdTAMs respectively recruit IFNγ-expressing CD4^+^ T cells to the tumour core, which in turn enhance T cell–recruiting chemokine production by TAMs.

Finally, we sought to determine whether TAMs have conserved chemotactic roles in human cancers. In Visium spatial transcriptomics of human colorectal tumours (36), there were hotspots enriched in monocyte/macrophage genes (including *CD14^+^CD68^+^*), and expression of *CXCL9* and *CXCL10* were localised to these regions (Fig 6L, Supplementary Fig S10A-D). We observed that CD4^+^ T-cell genes (including *TRAC^+^CD4^+^*) were enriched at these hotspots (Fig 6L, Supplementary Fig S10D-E). Since Visium slides capture at least 10 cells per 55 μm spot, we identified *CD14^+^CD68^+^* spots that also expressed *TRAC^+^CD4^+^* in the same spot (Fig 6M, Supplementary Fig S10F). We noted that ∼90% of these spots were *CXCL9/CXCL10*^+^ (Fig 6N, Supplementary Fig S10G-H). Hence, co-localisation of monocytes/macrophages and CD4^+^ T cells coincided with *CXCL9/CXCL10* expression in human tumours.

## Discussion

The phenotype and function of TAMs have been extensively investigated, but few studies have precisely tracked TAM fates following tumour entry in real-time. Using a photo-convertible murine model to site-specifically time-stamp cells, we have defined TAM subsets with distinct molecular profiles, spatio-temporal features, and functions. Importantly, we were able to demonstrate that mdTAMs form a highly dynamic pool of cells that undergo continual, rapid turnover. Following tumour entry, they swiftly differentiate down binary cell fate trajectories, losing inflammatory gene expression, but are amenable to activation by anti-PD-L1 therapy. These findings provide insights into TAM ontogeny, differentiation, subset-specific roles in tumour immunity and differential responses to ICB therapies.

Encroachment of tissue-resident macrophages or *de novo* recruitment of monocytes variably contribute to the macrophage compartment in solid tumours. *Ccr2*-deficient mice lacking monocyte precursors demonstrate incomplete depletion of TAMs in both spontaneous and transplanted tumour models (21,23,58), indicating the presence of a monocyte-independent TAM population. In MC38 tumours, both Ly6C^+^ and Ly6C^–^F4/80^high^ TAM subsets are affected by *Ccr2*-deficiency, but F4/80^high^ TAMs are better preserved (58). Indeed, studies have suggested that Ly6C^+^ monocytes may differentiate to F4/80^+^ TAMs (21,58), but pre-existing F4/80^+^ tissue macrophages may also contribute (24). We observed that F4/80^+^ rTAM were transcriptionally most similar to mdTAM_3, raising the possibility that rTAMs are the terminal state of the MHC-II^+^ mdTAM trajectory. Further application of progenitor-specific lineage tracing, or labelling approaches amenable to more prolonged cell tracking will be required to definitively address the origin of the “resident” TAMs. For example, a recent study utilised the inducible *Ms4a3-cre*^ERT2^ lineage reporter to track the fate of monocytes following tumour entry over several weeks (59). The authors found that monocytes terminally differentiate into two TAM populations, defined by *Trem2* or *Hif1a* expression, in a murine model of pancreatic adenocarcinoma.

Whether macrophage ontogeny influences functionality of TAMs remains largely unaddressed. In healthy tissues, it is postulated that the tissue environment has an important effect on macrophage function (60,61), but other studies demonstrate origin-dependent function (62). The spatial localisation of F4/80^high^ rTAMs at the tumour-normal interface and their interactions with PDGF-Rβ^+^ fibroblasts suggest that they have different roles to mdTAMs at the tumour core, for example, supporting ECM deposition (41). Indeed, depletion of TAMs from MC38 tumours led to aberrant ECM organisation (58). Similarly, in murine pancreatic adenocarcinomas, resident TAMs of embryonic origin express genes involved in ECM remodelling, and depletion of resident macrophages but not monocyte-derived macrophages reduced tumour progression (24). Importantly, deposition of a peri-tumoural fibrotic capsule may exclude T cells from the tumour (63), and increased density and organisation of peri-lesional ECM in colorectal tumours supports tumour invasion (64). Hence, the location and phenotype of TAMs may facilitate distinct roles in tumourigenesis.

Through the chronological sampling of tumours post-photoconversion, we have demonstrated that the emergence of binary MHC-II expression on mdTAMs occurs within hours following tumour entry. Upregulation of MHC-II in monocytes may be induced by GM-CSF (65) or IFNγ (66), which activate *Ciita* and *Rfx5* transcription factors (67,68) consistent with our transcriptomic analysis; but it is unclear whether other factors influence mdTAM fate. Here, we observed a higher proportion of MHC-II^+^ mdTAM in CT26 versus MC38 tumours, and an increase in anti-PD-L1-treated tumours, indicating that both tumour-intrinsic (69) and tumour-extrinsic factors influence this cell fate decision. MHC-II^+^ and MHC-II^–^ TAMs have been associated with tumour inhibition and progression respectively (70). Our data support that MHC-II^+^ mdTAMs may facilitate antibody-mediated antitumour responses (52) or activation of tumour-specific T cells through their capacity for antigen presentation (69,71). Of note, phagocytosis of tumour antigens by TAMs can directly induce expression of antigen presentation genes but leads to the simultaneous downregulation of pro-inflammatory effectors (72). We show that anti-PD-L1 treatment attenuates this loss of pro-inflammatory gene expression, and by skewing more mdTAM towards an immunogenic MHC-II^+^ state, may contribute to effective antitumour responses.

TAM states are variably associated with efficacy of ICB treatment (73), implying subset-specific roles in immunotherapy. We found that the transcriptional response to anti-PD-L1 treatment was most pronounced in newly-infiltrating mdTAM, with little effect on TAMs with prolonged tumour residence. In support of this observation, longitudinal sampling of murine sarcoma treated with anti-PD-1/anti-CTLA-4 suggested that macrophage activation following ICB was due to effects on circulating monocytes and early macrophages entering tumours, rather than mature intratumoural macrophages (74). Moreover, we noted subset-specific TAM activation signatures following anti-PD-L1, with skewing of incoming monocytes towards an MHC-II^+^ fate.

Activation of TAMs following anti-PD-L1 treatment has been attributed to increased IFNγ stimulation (75), and IFNγ-inducible CXCL9/10 production by TAMs following immune checkpoint therapy correlates with T-cell infiltration and is necessary for effective responses (76). Recent studies highlight the association between macrophage CXCL9/10 expression and response to immune checkpoint therapies in the clinic (77,78). Here, we report that rapid establishment of IFNγ-driven chemokine signalling following anti-PD-L1 is mediated by *de novo* influx of mdTAMs, as well as IFNγ- and CXCR3-expressing T cells. Interestingly, PD-L1-deficient macrophages exhibit aberrant development and function *in vitro*, including impaired IFNγ response (79), suggesting that PD-L1 may directly regulate macrophages by signalling in *cis* or reverse signalling. How this relates to anti-PD-L1 therapies in cancer, and whether cellular effects differ in anti-PD-1 therapy, will require further investigation.

One caveat to our study is that, although widely used in immune-oncology research (80), transplanted murine tumour models may variably recapitulate macrophage subsets and interactions in primary human cancers. Nevertheless, our study reveals functionally distinct TAM subsets that can be harnessed by ICB to differing extents, with important translational implications.

## Supplementary Material

Supplementary Table 1

Supplementary Figure

## Data Availability

The scRNA-seq data generated in this study are publicly available in Gene Expression Omnibus (GEO) at GSE221513 (https://www.ncbi.nlm.nih.gov/geo/query/acc.cgi?acc=GSE221513) and GSE221064 (https://www.ncbi.nlm.nih.gov/geo/query/acc.cgi?acc=GSE221064). The bulk RNA-seq data generated in this study are publicly available in GEO at GSE298881 (https://www.ncbi.nlm.nih.gov/geo/query/acc.cgi?acc=GSE298881). The scRNA-seq data from KPN, AKPT and *Apc*^fl/fl^ murine colon tumours is available at GSE280631 (https://www.ncbi.nlm.nih.gov/geo/query/acc.cgi?acc=GSE280631).

## Synopsis

The authors define tumour macrophage subsets with distinct molecular profiles, spatio-temporal features, and functions. These findings provide insights into tumour macrophage origin and differentiation, as well as subset-specific roles in tumour immunity and immune checkpoint inhibitor responses.
