# Supplementary material for: In vivo labelling resolves distinct temporal, spatial, and functional properties of tumour macrophages, and identifies subset-specific effects of PD-L1 blockade
Source: Cancer Immunol Res. Author manuscript; Available in PMC 2025 Jul 30. (PMC7617970; doi:10.1158/2326-6066.CIR-24-1233)
Supplement: Supplementary Table 1 [file EMS207524-supplement-Supplementary_Table_1.pdf]

Target	Fluorophore	Clone	Source	Application
CD11b	PE	M1/70	BioLegend	Microscopy
F4/80	AF488 / AF647	F4/80	Abcam	Microscopy
MHC Class II I-A/I-E	Pacific Blue	M5.114.15.2	BioLegend	Microscopy
CD3	AF488 / AF647	17A2	BioLegend	Microscopy
CD4	-	EPR19514	Abcam	Microscopy
CD31	AF594	MEC13.3	BioLegend	Microscopy
βIII-tubulin	-	ab18207	Abcam	Microscopy
ER-TR7	AF647	ER-TR7	Novus Biologicals	Microscopy
PDGFRb	PE	APB5	BioLegend	Microscopy
MPO	-	Polyclonal	Novus Biologicals	Microscopy
Donkey anti-rabbit IgG	AF647	Poylclonal	ThermoFisher	Microscopy
Donkey anti-goat IgG	AF488	Polyclonal	ThermoFisher	Microscopy
Donkey anti-goat IgG	AF594	Polyclonal	ThermoFisher	Microscopy
FcR Blocking reagent	-	-	Miltenyi Biotec	Flow
CD16/32	-	2.4G2	BioLegend	Flow
LIVE/DEAD™ Viability dye	APC-Cy7 / NIR	-	ThermoFisher	Flow
Viability dye	VK808	-	Beckman Coulter	Flow
CD45	BUV395 / BUV496 / BV785	30-F11	BioLegend	Flow
CD11c	AF647 / BV650	N418	Invitrogen	Flow
CD11b	BV785 / FITC	M1/70	BioLegend	Flow
Ly6C	BV605 / BV711	HK1.4	BioLegend	Flow
MHC Class II I-A/I-E	Pacific Blue / BV510	M5/114.15.2	BioLegend	Flow
PD-L1	PE-Cy7	10F.9G2	BioLegend	Flow
PD-L2/CD273	BUV395	TY25	BD Horizon	Flow
CD206	BV785	C068C2	BioLegend	Flow
CD64	BV421	X54-5/7.1	BioLegend	Flow
CD16	PE-Cy7	S17014E	BioLegend	Flow
CD16-2	PE	9E9	BioLegend	Flow
CD32b	APC	AT130-2	Invitrogen	Flow
CD80	BV605	16-10A1	BD	Flow
CD86	BV650	GL1	BD	Flow

CD40	FITC	HM40-3	ThermoFisher	Flow
F4/80	APC / PE-Cy7 / FITC	BM8	BioLegend	Flow
Ly6G	BV605 / APC-Cy7	1A8	BioLegend	Flow
CD3	BV650	17A2	BioLegend	Flow
B220	BV650	RA3-6B2	BioLegend	Flow
NK1.1	BV510 / BV650	PK136	BD Horizon	Flow
CXCL9	AF647	MIG-2F5.5	BioLegend	Flow
CXCL16	PE	12-81	BD	Flow
TNFa	PE	MP6-XT22	eBioscience	Flow
Ea52-68 peptide bound to I-Ab	Biotinylated	eBioY-Ae (Y-Ae)	Invitrogen	Flow
Streptavidin	PE-Cy7	-	BioLegend	Flow
Ovalbumin (protein)	AF647	-	Invitrogen	Flow
CD45	BV785	30-F11	BioLegend	FACS
CD11b	eFluor 450	M1/70	Invitrogen	FACS
Ter119	PE-Cy7	TER-119	Invitrogen	FACS
LIVE/DEAD™ Viability/Cytotoxicity dye	APC-Cy7	-	ThermoFisher	FACS
NK1.1	BV650	PK136	BD	FACS
