# Supplementary material for: In vivo labelling resolves distinct temporal, spatial, and functional properties of tumour macrophages, and identifies subset-specific effects of PD-L1 blockade
Source: Cancer Immunol Res. Author manuscript; Available in PMC 2025 Jul 30. (PMC7617970; doi:10.1158/2326-6066.CIR-24-1233)
Supplement: Supplementary Figure [file EMS207524-supplement-Supplementary_Figure.pdf]

Supplementary figure 1

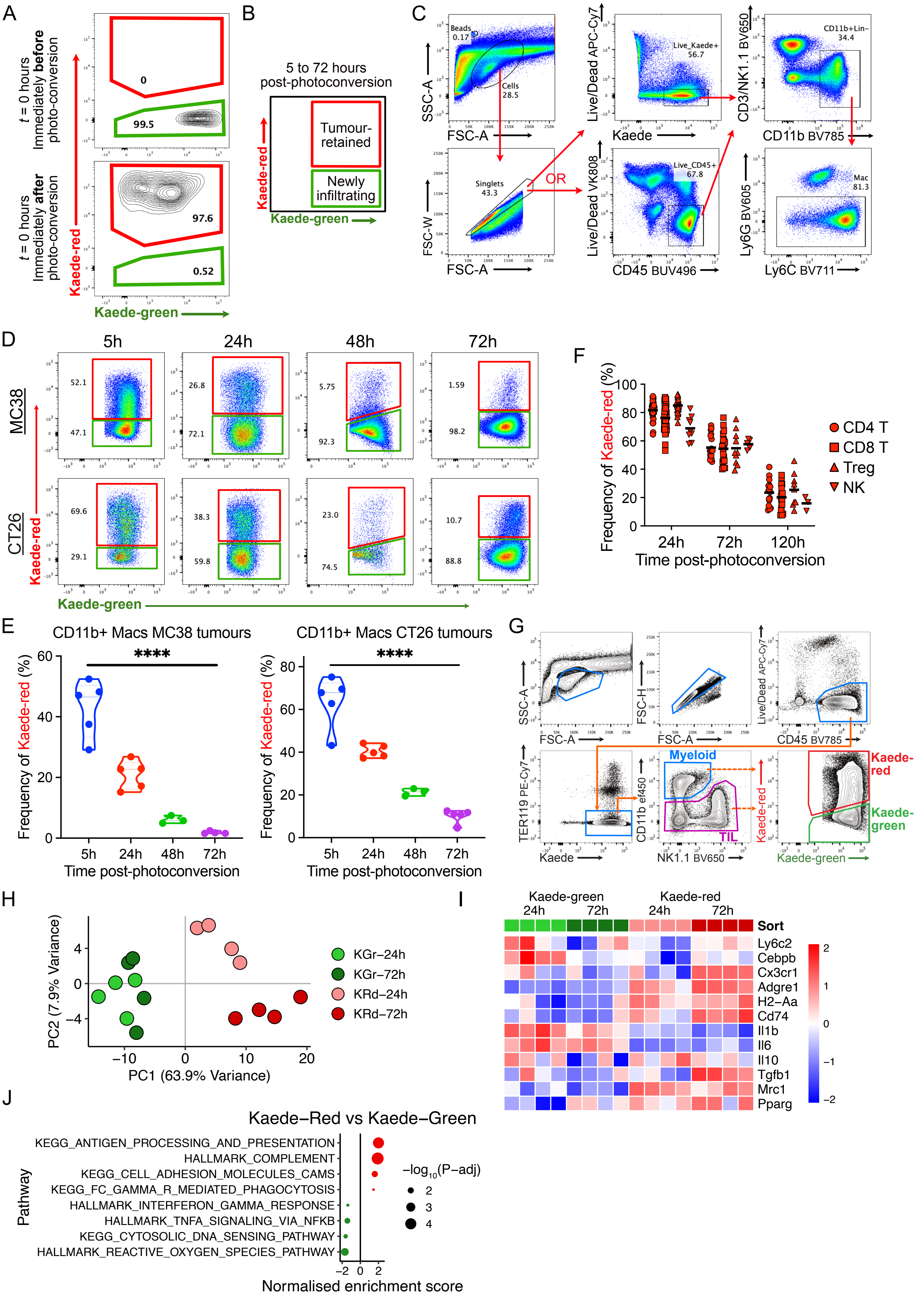

Supplementary figure 1

(A) Flow cytometry of Kaede-green and Kaede-red cells immediately prior to and following photo-conversion. Mean photoconversion efficiency is 98%; all experiments achieved >95% photoconversion efficiency.

(B) Schematic of flow cytometry for identification of newly-infiltrating (Kaede-green) or tumour-retained (Kaede-red) cells after photo-conversion.

(C) Flow gating strategy for identification of tumour-associated macrophages (TAM). Flow cytometry gating (A-C) apply to all tumour models used in this study.

(D) Representative flow cytometry of Kaede fluorescence of lineage^{-}CD11b^{+} cells 5 to 72h post-photoconversion from subcutaneous MC38 and CT26 tumours.

(E) Quantification of (D). One-way ANOVA test was used.

(F) Frequency of Kaede-red cells among CD4 T, CD8 T, regulatory T, and NK cells in MC38 tumours post-photoconversion. Points represent tumours from independent mice (E-F).

(G) FACS sort strategy for isolation of live TER119^{-}CD45^{+}Kaede^{+} MC38-Ova tumour-infiltrating immune cells; cells were sorted to 'myeloid' and 'TIL' populations and by Kaede-fluorescence.

(H) Principal component analysis (PCA) of bulk RNA-seq of lineage^{-}CD11b^{+} Kaede-green/red cells 24h or 72h post-photoconversion. Points represent tumours from independent mice.

(I) Heatmap (z-scores) of selected gene expression from bulk RNA-seq in (H).

(J) Gene set enrichment analysis (GSEA) of Kaede-green versus Kaede-red lineage^{-}CD11b^{+} cells. Bulk RNA-seq data (H-J) are from MC38-Ova tumours.

Supplementary figure 2

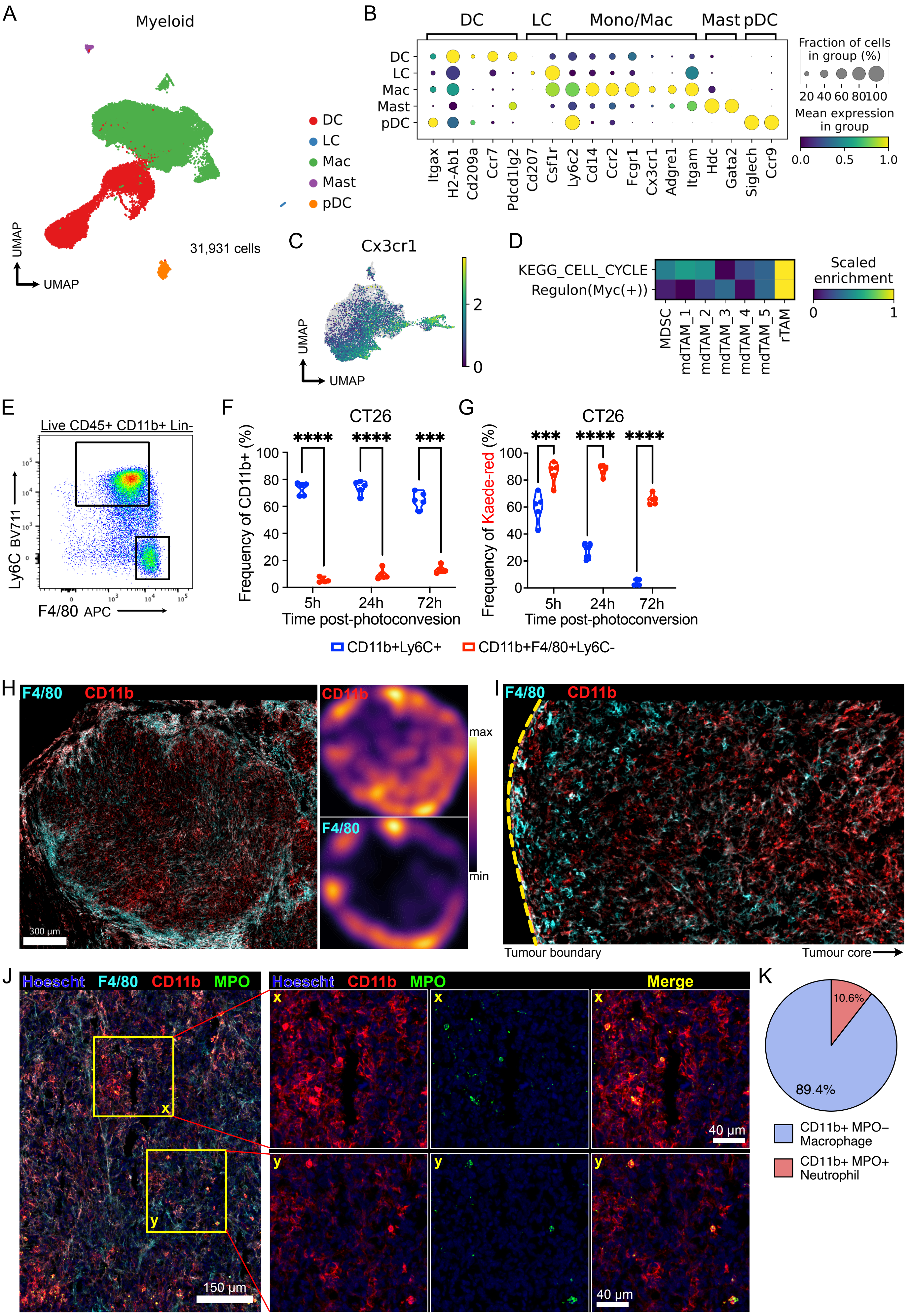

Supplementary figure 2

(A) Uniform Manifold Approximation and Projection (UMAP) of scRNA-seq of myeloid cells from tumour-infiltrating immune cells.
(B) Selected marker gene expression of clusters from (A).
(C) *Cx3cr1* expression.
(D) Enrichment of 'KEGG cell cycle' genes and Myc regulon activity scores.
(E) Flow gating strategy for identification of TAM subsets by Ly6C and F4/80 expression.
(F-G) Flow cytometry of frequency of TAM subsets (F) and their Kaede fluorescence (G) from CT26 tumours (related to Fig 1H) 5h to 72h post-photoconversion. Paired t-test with FDR correction was used; points represent tumours from independent mice.
(H) Independent representative IF microscopy image of $CD11b^{+}F4/80^{+}$ macrophages in MC38 tumours and kernel density heatmaps for staining of selected markers.
(I) Zoomed-in representative IF microscopy image of F4/80 and CD11b expression.
(J) IF microscopy of Hoechst (nuclei), F4/80, CD11b and MPO (neutrophils) expression in MC38 tumours with high-resolution insets labelled '**x**' and '**y**'.
(K) Quantification of MPO expression in $CD11b^{+}$ cells from imaging analysis of whole tumour sections.

Supplementary figure 3

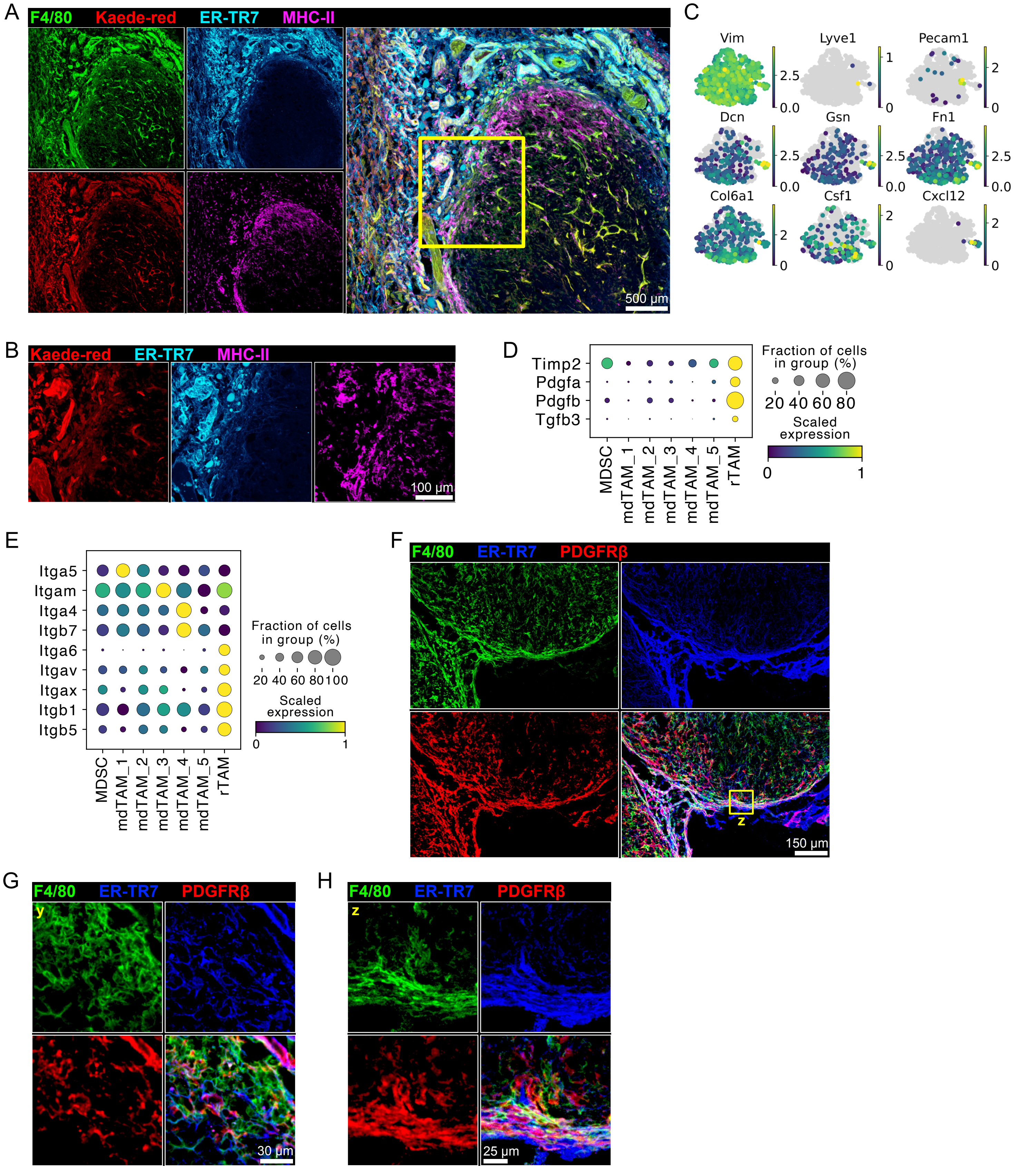

Supplementary figure 3

(A) IF microscopy of Kaede-fluorescence, F4/80, MHC-II and ER-TR7 (fibroblasts/stroma) expression in subcutaneous MC38 tumours, with zoomed-in inset (yellow box, B).
(C) UMAP of scRNA-seq of stromal cells (fibroblasts), showing selected gene expression.
(D-E) Selected gene expression in TAM clusters.
(F) Independent representative IF microscopy of macrophages and fibroblasts in MC38 tumours.
(G) High-resolution zoomed-in section of inset labelled '**y**' from Fig 2F.
(H) High-resolution zoomed-in section of inset labelled '**z**' from (F).

Supplementary figure 4

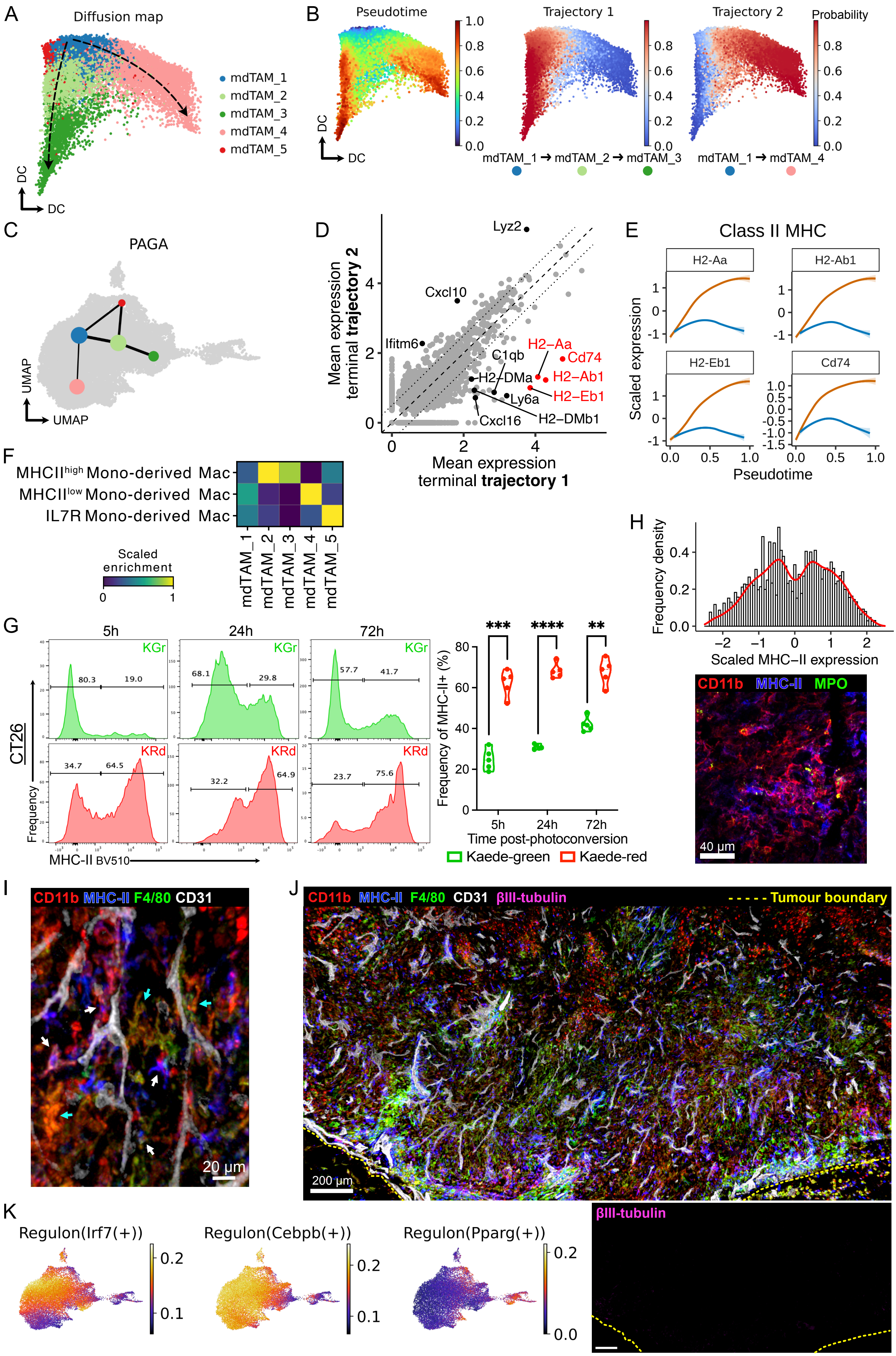

Supplementary figure 4

(A) Diffusion map embedding of scRNA-seq of mdTAMs. Arrows indicate proposed maturation trajectories.

(B) Palantir pseudotime analysis. Colour in left panel represents pseudotime values, and right panel represents probability of a cell belonging to the specified trajectory.

(C) PAGA analysis of mdTAMs.

(D) Mean expression of genes in trajectory termini (trajectory probability > 0.7). Selected differentially expressed genes (DEG) between trajectories are labelled, MHC-II genes in red.

(E) Expression of class II MHC genes across pseudotime by mdTAM trajectory.

(F) Enrichment of published macrophage subset-specific gene signatures (GSE125691, E-MTAB-8207).

(G) Representative flow cytometry of MHC-II expression on lineage$^-$CD11b$^+$Ly6C$^+$ mdTAMs, by Kaede fluorescence, 5h to 72h post-photoconversion in CT26 tumours; and quantification. Paired t-test with FDR correction was used; points represent tumours from independent mice.

(H) Histogram and density plot of MHC-II expression on CD11b$^+$MPO$^-$ macrophages within the tumour core, and accompanying IF microscopy section demonstrating CD11b$^+$MPO$^-$MHC-II$^+$ and CD11b$^+$MPO$^-$MHC-II$^-$ macrophages.

(I) Independent IF microscopy section of CD11b$^+$F4/80$^{+/-}$MHC-II$^+$ macrophages (white arrows) and CD11b$^+$F4/80$^{+/-}$MHC-II$^-$ macrophages (cyan arrows) with CD31$^+$ blood vessels in MC38 tumours.

(J) IF microscopy of CD11b, MHC-II, F4/80, CD31 (blood vessels) and βIII-tubulin (nerves) in MC38 tumours. Representative of 3 independent experiments (I-J).

(K) Selected regulon activity scores differentially enriched in mdTAMs versus rTAMs.

Supplementary figure 5

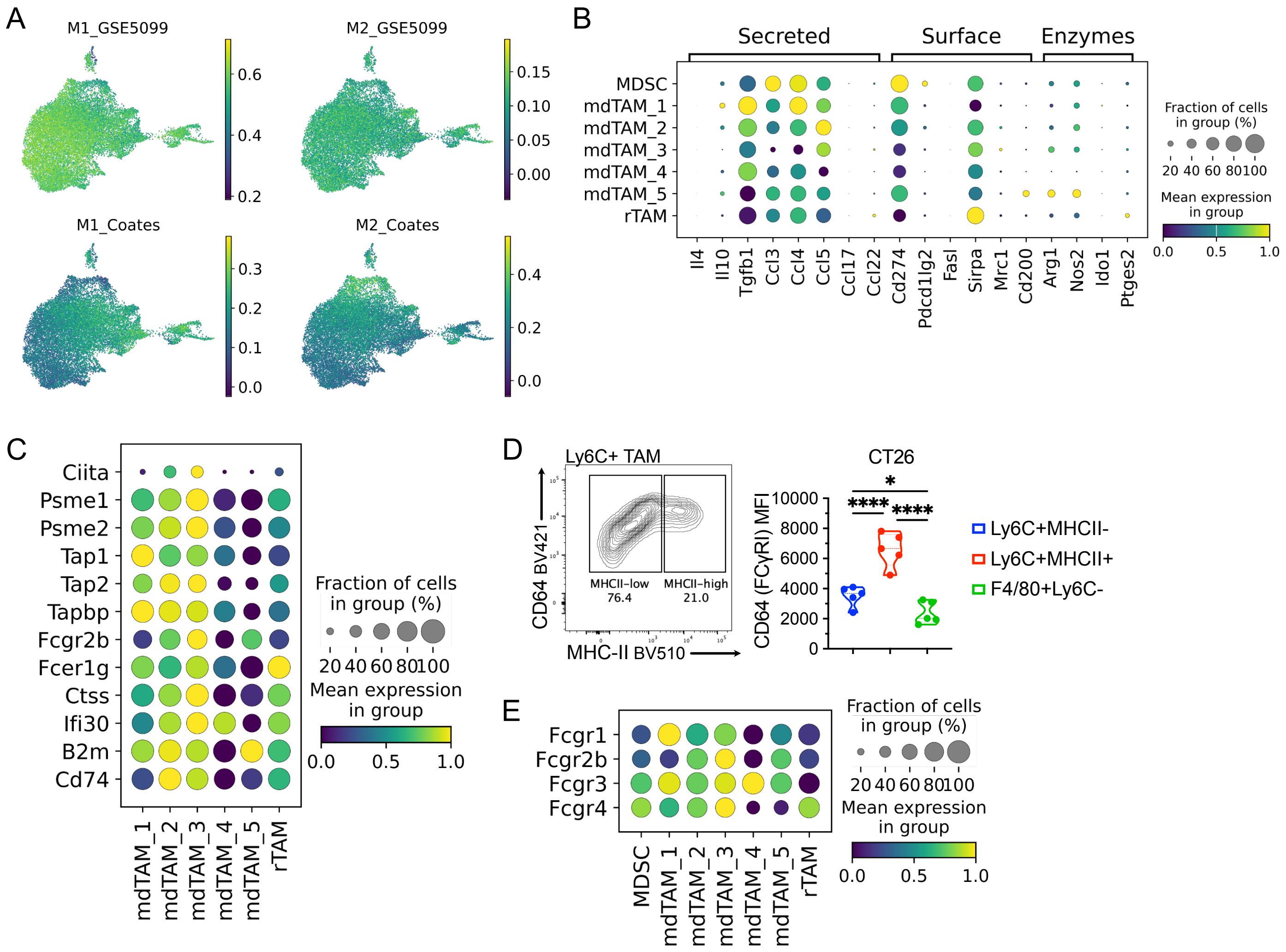

Supplementary figure 5

(A) Enrichment of “M1” and “M2” macrophage gene signatures from MSigDB.

(B) Expression of immunosuppressive molecules (3).

(C) Expression of selected genes from ‘KEGG antigen processing and presentation’.

(D) Representative flow cytometry of surface FcγRI expression in MC38 tumours and quantification of FcγRI expression in CT26 tumours.

(E) Expression of FcγR genes.

Supplementary figure 6

Villin-cre^{ERT2} *Kras*$^{G12D/+}$ *Trp53*$^{fl/fl}$ *Rosa26*$^{N1icd/+}$(*Notch1*^{Tg}) (**KPN**) colorectal tumours
Orthotopic tumour organoid transplantation

A

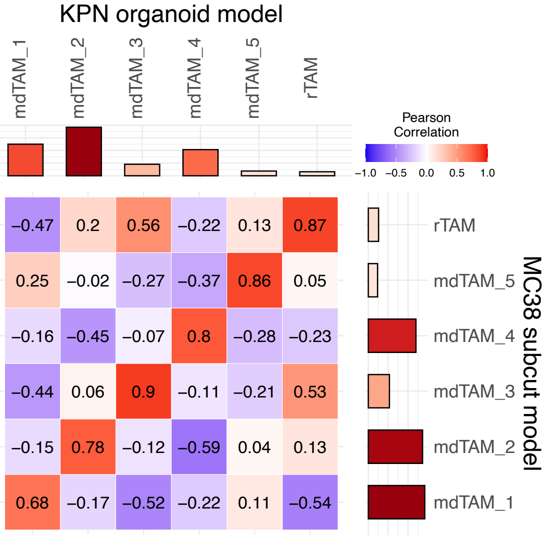

B

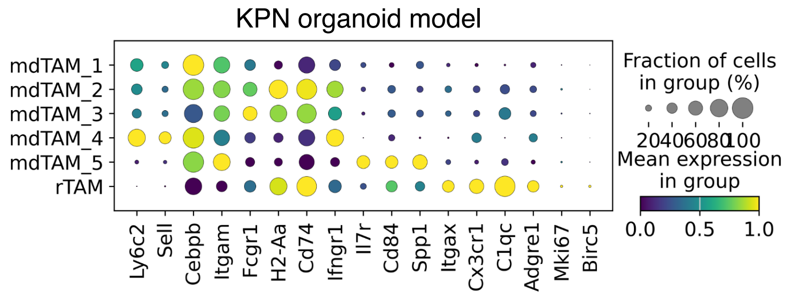

Villin-cre^{ERT2} *Apc*$^{fl/fl}$ *Kras*$^{G12D/+}$ *Trp53*$^{fl/fl}$ *Tgbr1*$^{fl/fl}$ (**AKPT**) colorectal tumours
Orthotopic tumour organoid transplantation

C

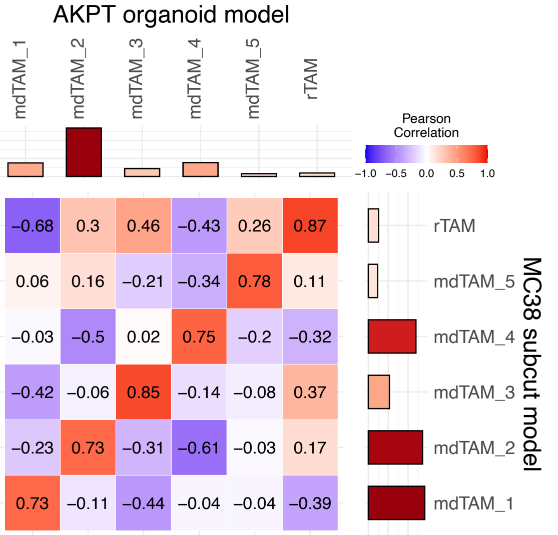

D

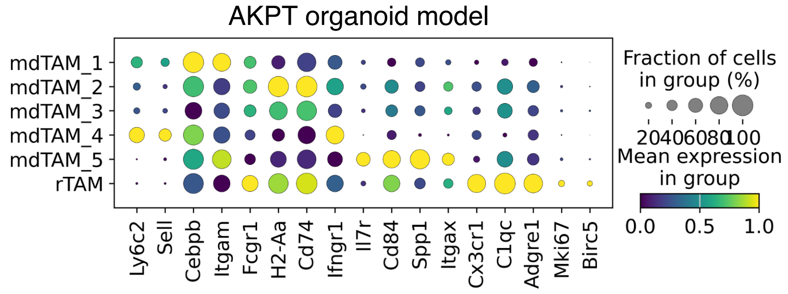

Villin-cre^{ERT2} *Apc*$^{fl/fl}$ colorectal tumours
Tamoxifen induction model

E

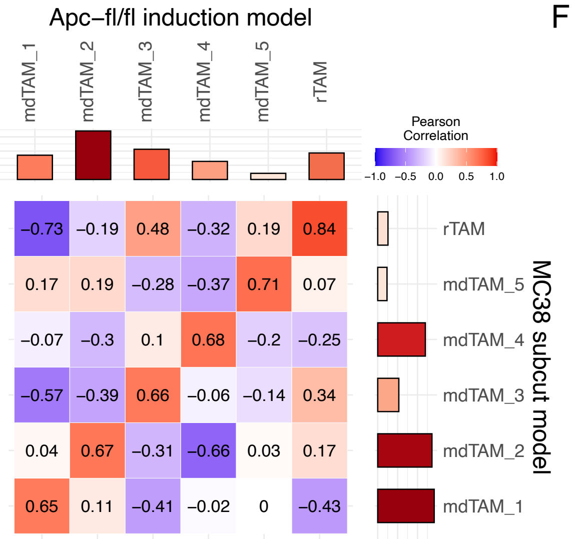

F

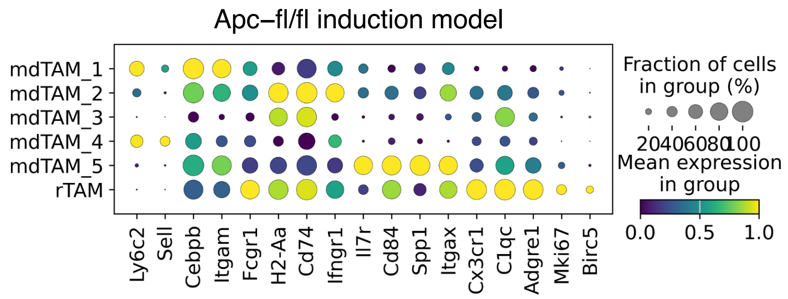

Supplementary figure 6

(A) Pearson correlation of TAM clusters from scRNA-seq of orthotopically transplanted *Kras*$^{G12D/+}$ *Trp53*$^{fl/fl}$ *Rosa26*$^{N1icd/+}$(*Notch1*^{Tg}) (KPN) colon tumour organoids and MC38 tumours, following integration and label transfer. Bars indicate relative proportions of each cell state in the respective models.
(B) Selected gene expression in TAMs from KPN tumours.
(C) Pearson correlation of TAM clusters from scRNA-seq of orthotopically transplanted *Apc*$^{fl/fl}$ *Kras*$^{G12D/+}$ *Trp53*$^{fl/fl}$ *Tgfbr1*$^{fl/fl}$ (AKPT) colon tumour organoids and MC38 tumours.
(D) Selected gene expression in TAMs from AKPT tumours.
(E) Pearson correlation of TAM clusters from scRNA-seq of Tamoxifen-induced *Villin-cre*^{ERT2} *Apc*$^{fl/fl}$ colon tumours and MC38 tumours.
(F) Selected gene expression in TAMs from *Apc*$^{fl/fl}$ tumours.

Supplementary figure 7

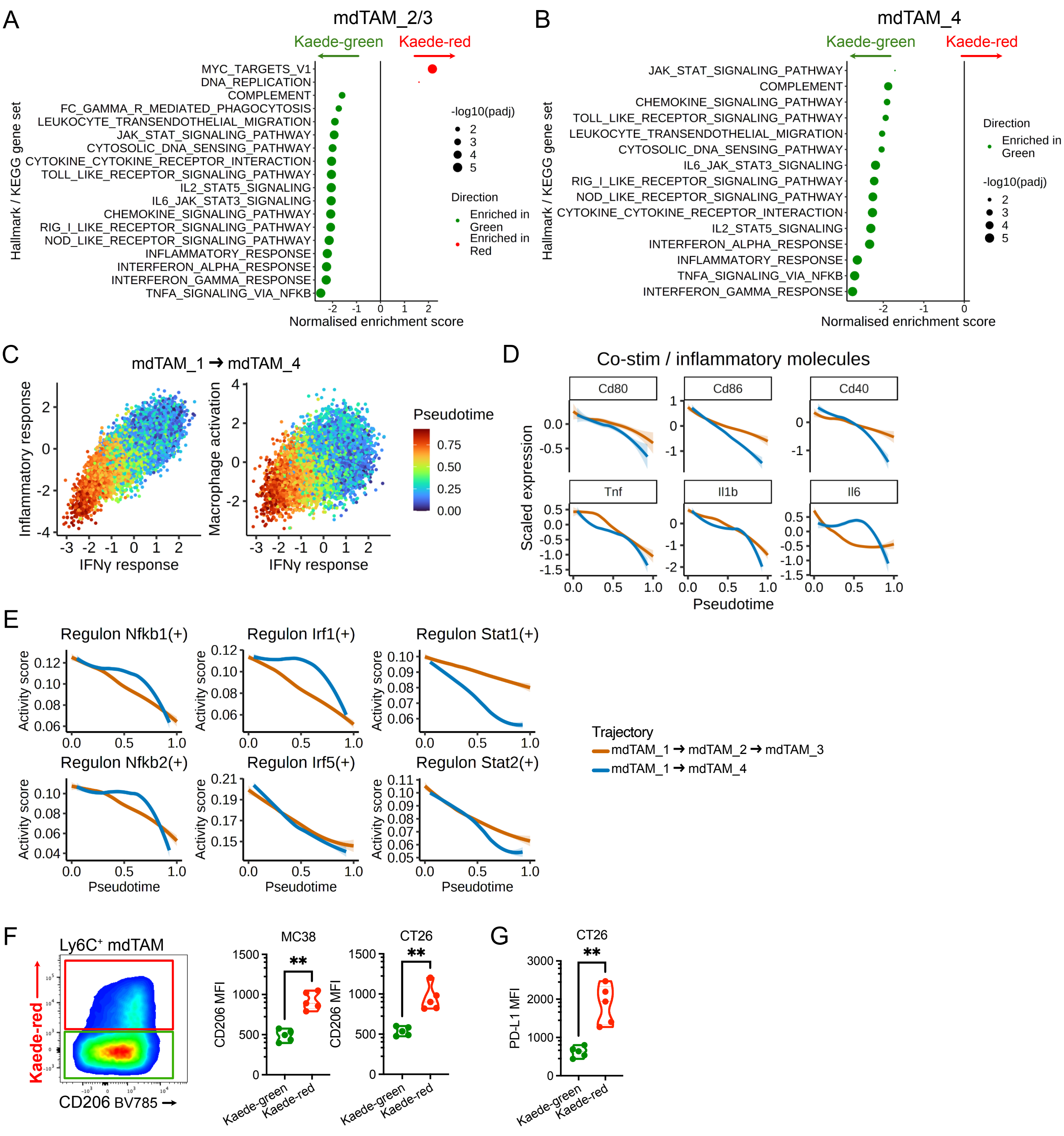

Supplementary figure 7

(A-B) GSEA of scRNA-seq of Kaede-red versus Kaede-green cells in mdTAM_2/3 and mdTAM_4.
(C) Scatterplot of cells from the mdTAM_1-to-mdTAM_4 trajectory, showing scaled gene set enrichment (x/y axis) over pseudotime (colour).
(D) Expression of selected co-stimulatory molecules and cytokines across pseudotime.
(E) Enrichment scores for selected regulon activity scores across pseudotime.
(F) Flow cytometry of surface CD206 expression in $Ly6C^+$ TAMs 24h post-photoconversion.
(G) Flow cytometry of surface PD-L1 expression in Kaede-red versus Kaede-green $Ly6C^+$ TAMs 24h post-photoconversion, from CT26 tumours. Paired t-tests were used; points represent tumours from independent mice (F-G).

Supplementary figure 8

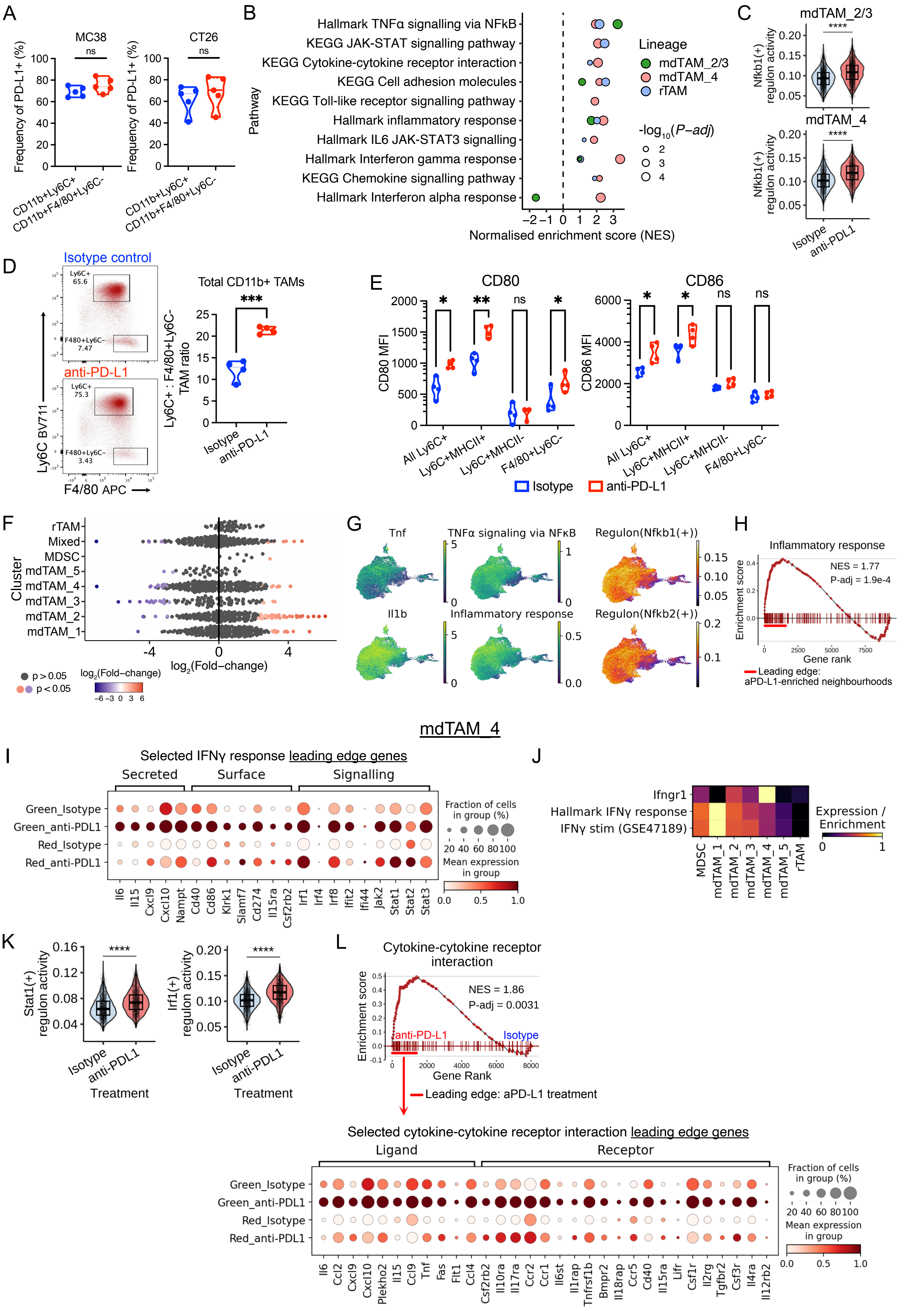

Supplementary figure 8

(A) Expression of PD-L1 in TAM subsets in MC38 and CT26 tumours. Paired t-test was used.
(B) GSEA of Hallmark and KEGG gene sets in TAMs from anti-PD-L1-treated (NES > 0) versus isotype control-treated (NES < 0) tumours, by TAM cluster/trajectory.
(C) *Nfkb1* regulon activity score in mdTAM clusters.
(D) Frequency (ratio) of $Ly6C^{+}$ mdTAM to $F4/80^{+}Ly6C^{-}$ rTAM in anti-PD-L1-treated versus isotype control-treated MC38 tumours *in vivo*.
(E) Expression of CD80 and CD86 across TAM subsets following treatment. Student's t-tests with FDR correction were used; points represent tumours from independent mice (A,D-E).
(F) Milo differential abundance analysis of TAMs.
(G) Expression or enrichment of selected genes, gene sets, or regulon activity scores.
(H) GSEA of selected neighbourhoods encircled in Fig 5J versus non-anti-PD-L1 enriched Kaede-red majority (>60%) neighbourhoods in mdTAM_2/3.
(I) Leading-edge genes from GSEA of 'Hallmark IFNγ response' in mdTAM_4.
(J) Expression of *Ifngr1* and enrichment of IFNγ-response genesets.
(K) *Stat1* and *Irf1* regulon activity scores in mdTAM_4. Mann-Whitney U test was used (C, K).
(L) GSEA of 'KEGG cytokine-cytokine receptor interaction' in mdTAM_4, comparing anti-PD-L1 versus isotype control-treated tumours, and leading-edge genes.

Supplementary figure 9

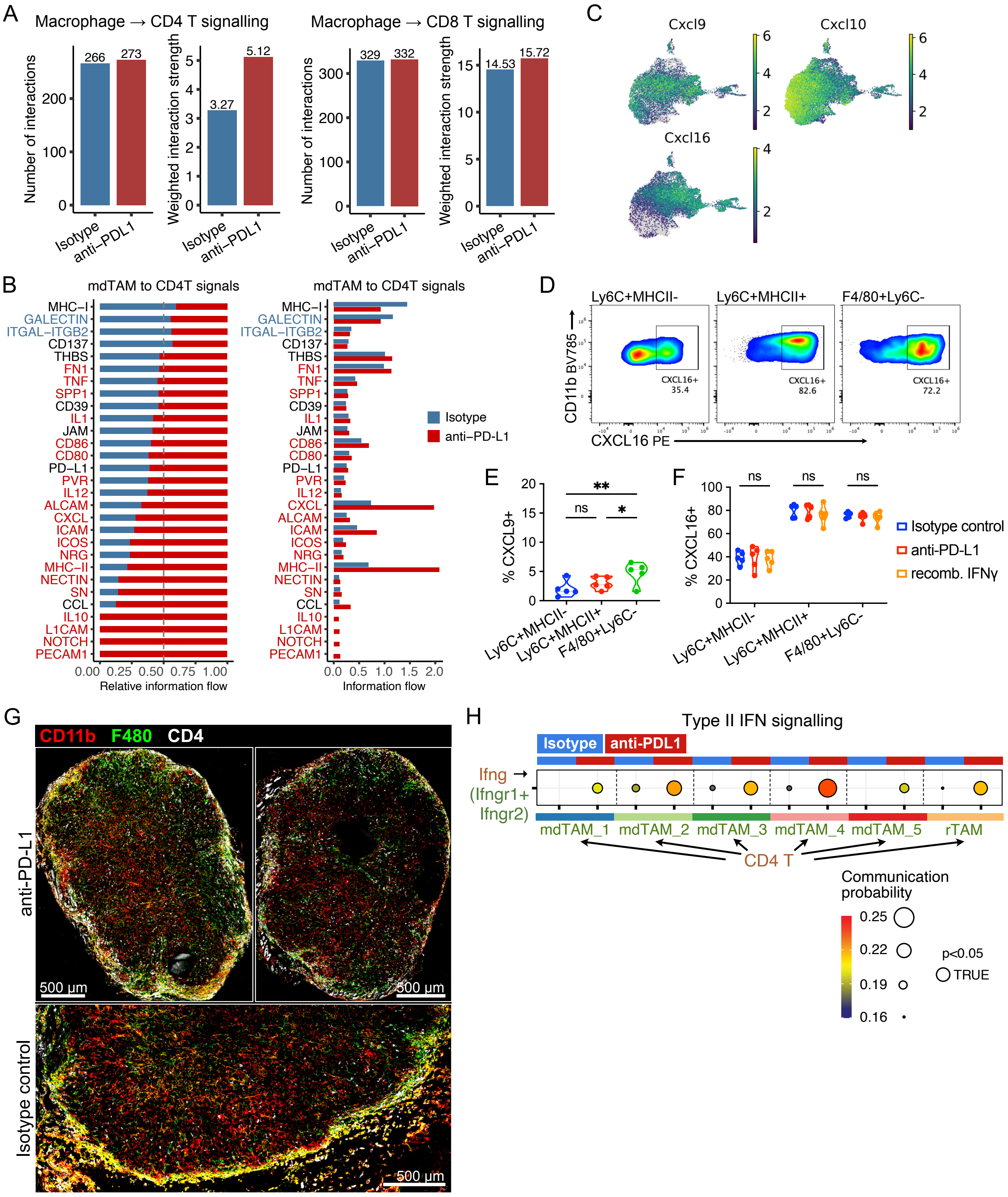

Supplementary figure 9

(A) Cell-cell communication analysis of scRNA-seq of macrophages and $CD4^+$ or $CD8^+$ T cells in anti-PD-L1 treated versus isotype control-treated tumours.

(B) Differential signal strengths from mdTAM to $CD4^+$ T cells, by signalling pathway. Coloured pathways (text) are significantly different.

(C) Expression of *Cxcl9*, *Cxcl10* and *Cxcl16*.

(D) Representative flow cytometry of intracellular CXCL16 expression in TAMs from unstimulated tumours.

(E) Flow cytometry of intracellular CXCL9 expression in TAMs from unstimulated tumours. One-way ANOVA with Šidák's multiple comparisons test was used.

(F) Flow cytometry of intracellular CXCL16 expression in TAMs from tumours treated with isotype control antibodies, anti-PD-L1 antibodies, or recombinant IFNγ *ex vivo* for 8h. Paired t-test with FDR correction was used. Points represent tumours from independent mice (E-F).

(G) IF microscopy of TAMs and $CD4^+$ T cells from isotype control or anti-PD-L1-treated tumours. Data is representative of 3 independent experiments.

(H) Ligand-receptor interactions between $CD4^+$ T cells and TAMs from type II IFN signalling pathway.

Supplementary figure 10

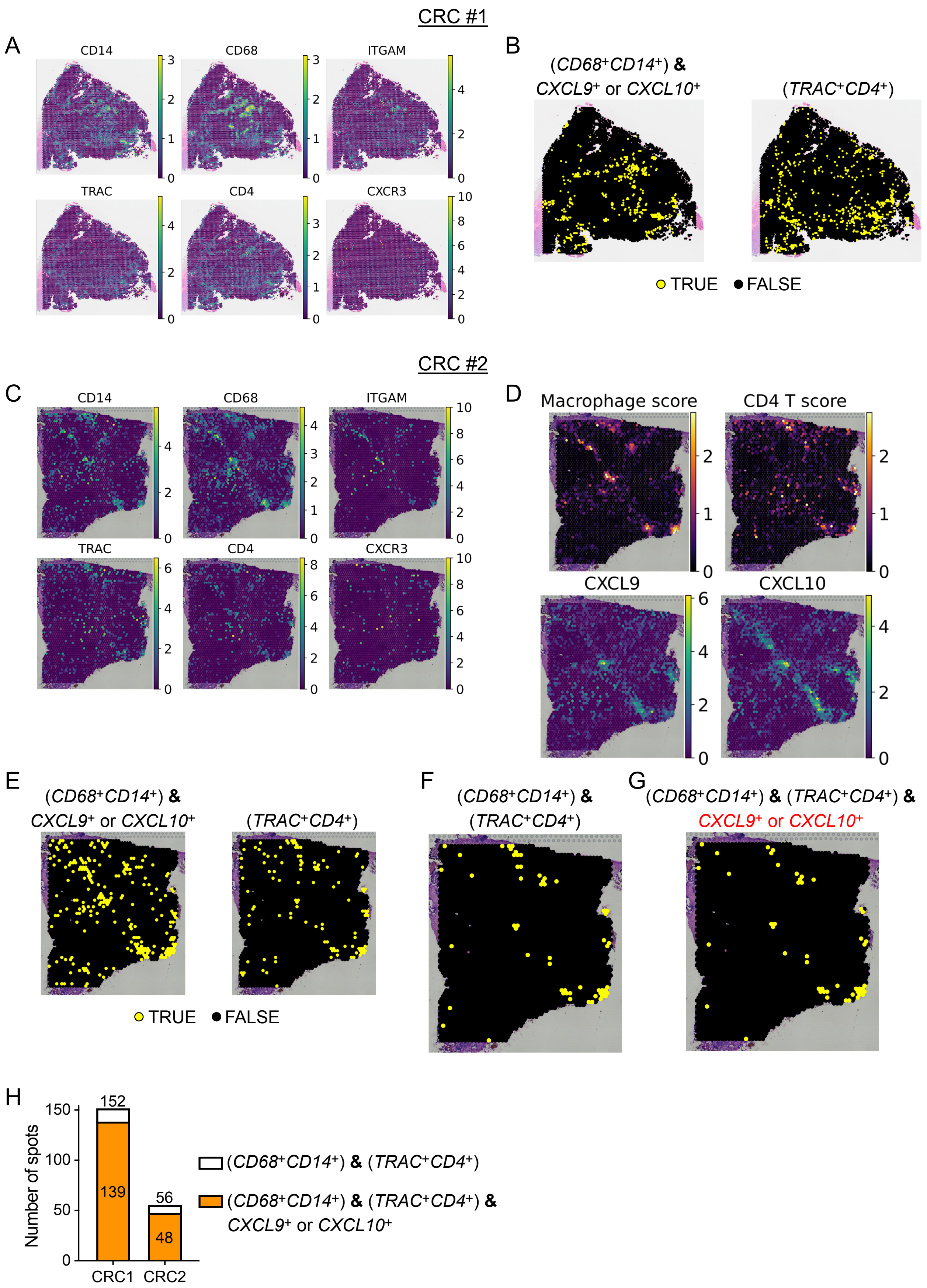

Supplementary figure 10
(A-B) Supporting data for Visium (10X Genomics) spatial transcriptomics data from Fig 6L-N (CRC #1; AJCC: stage II-A; TNM: T3), and (C-F) Visium (10X Genomics) spatial transcriptomics data of an independent human colorectal tumour (CRC #2; AJCC: stage II-B; TNM: T4N0M0); *n* = 2.
(A) Selected gene expression in CRC #1.
(B) Left panel, spots with expression of *CD68* and *CD14* (monocytes/macrophages) and CXCL9 or CXCL10; and right panel, spots with expression of *TRAC* and *CD4* ($CD4^+$ T cells).
(C) Selected gene expression in CRC #2.
(D) Macrophage and $CD4^+$ T cell gene enrichment and CXCL9/10 expression.
(E) Left panel, spots with expression of *CD68* and *CD14* (monocytes/macrophages) and CXCL9 or CXCL10; and right panel, spots with expression of *TRAC* and *CD4* ($CD4^+$ T cells).
(F) Spots with expression of *CD68* and *CD14* (monocytes/macrophages), and *TRAC* and *CD4* ($CD4^+$ T cells).
(G) Spots fulfilling conditions in (F) and expressing *CXCL9* or *CXCL10*.
(H) Quantification of Fig 6N and S10G.
